# Therapeutic Potential of *Lythrum salicaria* L. Ethanol Extract in Experimental Rat Models of Streptozotocin-Induced Diabetes Mellitus and Letrozole-Induced Polycystic Ovary Syndrome

**DOI:** 10.3390/antiox14050573

**Published:** 2025-05-10

**Authors:** Lia Oxana Usatiuc, Marcel Pârvu, Raluca Maria Pop, Ana Uifălean, Dan Vălean, Adrian Surd, Mădălina Țicolea, Ana Hîruța, Floricuța Ranga, Florinela Adriana Cătoi, Corina Cătană, Alina Elena Pârvu

**Affiliations:** 1 Pathophysiology, Department 2—Functional Sciences, Faculty of Medicine, “Iuliu Hațieganu” University of Medicine and Pharmacy, 400012 Cluj-Napoca, Romania; lia.usatiuc@umfcluj.ro (L.O.U.); ana.uifalea@umfcluj.ro (A.U.); madalina.ticolea@umfcluj.ro (M.Ț.); adriana.catoi@umfcluj.ro (F.A.C.); parvualinaelena@umfcluj.ro (A.E.P.); 2 Department of Taxonomy, Faculty of Biology and Geology, “Babes-Bolyai” University, 400012 Cluj-Napoca, Romania; marcel.parvu@ubbcluj.ro; 3 Pharmacology, Toxicology and Clinical Pharmacology, Department 2—Functional Sciences, Faculty of Medicine, “Iuliu Haţieganu” University of Medicine and Pharmacy, 400012 Cluj-Napoca, Romania; 4 Surgery Department, “Iuliu Haţieganu” University of Medicine and Pharmacy, 400012 Cluj-Napoca, Romania; valean.dan@elearn.umfcluj.ro; 5 Pediatric Surgery and Orthopedics, Department of Mother and Child, “Iuliu Hatieganu” University of Medicine and Pharmacy, 400012 Cluj-Napoca, Romania; adisurd@elearn.umfcluj.ro; 6 Pathology Department, University of Agricultural Sciences and Veterinary Medicine of Cluj-Napoca, 400372 Cluj-Napoca, Romania; ana.hiruta@usamvcluj.ro; 7 Food Science and Technology, Department of Food Science, University of Agricultural Science and Veterinary Medicine of Cluj-Napoca, 400372 Cluj-Napoca, Romania; florica.ranga@usamvcluj.ro; 8 Center for Biodiversity and Conservation, Faculty of Horticulture and Business in Rural Development, University of Agricultural Sciences and Veterinary Medicine of Cluj-Napoca, 400372 Cluj-Napoca, Romania; corina.catana@usamvcluj.ro

**Keywords:** *Lythrum salicaria* L., diabetes mellitus, polycystic ovary syndrome, oxidative stress, inflammation, insulin resistance

## Abstract

Polycystic ovary syndrome (PCOS) and diabetes mellitus (DM) are prevalent endocrine disorders with overlapping pathophysiological mechanisms. Type 2 diabetes mellitus (T2DM) is commonly associated with PCOS, with both conditions strongly linked to insulin resistance (IR), while recent studies have also reported an increased prevalence of PCOS among women with type 1 diabetes mellitus (T1DM). This study evaluated the potential of *Lythrum salicaria* L. ethanol extract (LSEE) to mitigate oxidative stress (OS), inflammation, and metabolic and hormonal imbalances in separate experimental models of Streptozotocin (STZ)-induced DM and Letrozole (LET)-induced PCOS. LSEE underwent phytochemical analysis to quantify total phenolic and flavonoid content and HPLC-MS for polyphenols identification. In vitro, antioxidant capacity was investigated through FRAP, DPPH, NO, and H_2_O_2_ scavenging assays. Subsequently, in vivo, studies utilized STZ-induced DM and LET-induced PCOS rat models, with 10-day treatments of LSEE, metformin, or trolox (TX) administered by gavage. Dysregulation of hormonal profiles, ultrasound, and histological examinations confirmed PCOS development. At the end of the treatment period, serum samples were collected to assess OS markers (TOS, OSI, MDA, AOPP, 8-OHdG, NO, 3-NT, AGEs, TAR, SH) in both models. Inflammatory markers were also measured (IL-1β, NF-κB, IL-18, and Gasdermin D in DM and IL-1β, NF-κB, IL-18, and IL-10 in PCOS). Additionally, metabolic markers (glucose, lipids, TG-glucose index, liver enzymes) were assessed in DM rats, and hormones (LH, FSH, estrogen, testosterone, insulin, HOMA-IR) were determined in PCOS rats. LSEE demonstrated a high polyphenolic content and notable in vitro antioxidant activity. In vivo, it effectively reduced OS by lowering oxidant levels and enhancing antioxidant defenses, reduced inflammatory markers and blood glucose levels, and improved lipid profiles along with the TyG index and liver injury markers in diabetic rats. In PCOS rats, LSEE lowered the total oxidants, increased antioxidants, reduced LH, FSH, testosterone, and insulin, and increased estrogen levels. The effects exhibited a dose-dependent pattern, with higher doses producing more pronounced benefits comparable to those observed with metformin and TX. In conclusion, LSEE may be a promising complementary treatment for DM and PCOS.

## 1. Introduction

Diabetes mellitus (DM) represents a chronic metabolic disorder marked by impaired glucose regulation, resulting from either absolute or relative insulin deficiency, encompassing both type 1 (T1DM) and type 2 (T2DM) diabetes. Its prevalence has risen dramatically over recent decades, causing a major public health challenge with devastating complications [1].

Polycystic ovary syndrome (PCOS) is a heterogeneous endocrine disorder characterized by ovulatory dysfunction, hyperandrogenism, and polycystic ovarian morphology. Its pathophysiology is complex and multifactorial, involving dysregulation of the hypothalamic–pituitary–ovarian axis, insulin resistance (IR), and genetic predisposition. PCOS affects an estimated 5–15% of women of reproductive age worldwide [2,3].

PCOS and DM, particularly type 2 DM, exhibit a complex bidirectional relationship characterized by shared pathophysiological mechanisms, with IR serving as a central link. In patients with T1DM, delayed puberty in adolescents and a higher prevalence of hirsutism and PCOS features have been observed. It is hypothesized that exogenous insulin therapy in T1DM, which bypasses hepatic first-pass metabolism and leads to elevated systemic insulin levels, may contribute to ovarian hyperandrogenism by stimulating theca cells androgen direct or indirect release [4,5]. Insulin has been shown to act as a co-gonadotropin, enhancing steroidogenic responses to gonadotropins and promoting ovarian cyst formation in experimental models. Furthermore, insulin suppresses hepatic production of sex hormone-binding globulin (SHBG), thereby increasing free testosterone levels. These mechanisms suggest a potential link between hyperinsulinemia in T1DM and PCOS-like symptoms, supporting the relevance of exploring ovarian effects in diabetic models.

Oxidative stress (OS), characterized by an excess of reactive oxygen species (ROS), is a key contributor to DM and PCOS. In DM, hyperglycemia induces OS, which further leads to pro-inflammatory pathways, causing a vicious pathogenic cycle that promotes the development of complications. In PCOS, OS disrupts steroidogenesis, causing androgen excess, impairs insulin signaling, leading to IR, and promotes the chronic low-grade inflammation commonly observed in PCOS. As a result, OS likely explains the increased risk of DM in women with PCOS, with OS-driven IR and inflammation acting as shared pathogenic mechanisms [6].

Persistent, low-grade inflammation is now understood to be a core component in the development of DM, especially T2DM. This chronic inflammation fuels IR and drives the progression of diabetic complications. The NLRP3 inflammasome, a key inflammatory mediator, plays a critical role in DM pathogenesis [7]. Likewise, in PCOS, inflammation is not merely a by-product of metabolic abnormalities but a direct contributor to the PCOS pathophysiology and associated comorbidities. It exacerbates IR, increases androgen levels, impairs ovulation, and elevates the risk of metabolic complications in women with PCOS [8,9].

*Lythrum salicaria* L. (purple loosestrife) is a perennial herb traditionally used in herbal medicine, particularly in Europe. Historical ethnomedicinal records show that *L. salicaria* L. was widely employed as a remedy for gastrointestinal ailments, especially dysentery and chronic diarrhea, as well as for various inflammatory conditions of the skin and mucous membranes. Phytochemical analyses revealed that *L. salicaria* L. is rich in polyphenolic compounds and polysaccharides [1]. In particular, ellagitannins and flavonoid glycosides have been identified as dominant constituents of the herb. Extracts of *L. salicaria* L. have demonstrated a broad spectrum of bioactivities, including pronounced antioxidant and anti-inflammatory effects, as well as antimicrobial, antidiarrheal, and even anti-diabetic properties [10,11].

Despite the well-documented antioxidant and anti-inflammatory properties of *L. salicaria* L., its therapeutic potential in complex metabolic-endocrine disorders such as DM and PCOS remains poorly explored, particularly in the context of dual oxidative and inflammatory stress models. Therefore, our study aimed to fill this critical knowledge gap by investigating the effects of LSEE on OS and inflammation, and their downstream influence on metabolic and hormonal dysfunctions. Specifically, we evaluated LSEE’s efficacy in modulating glucose and lipid metabolism in a streptozotocin (STZ)-induced diabetic rat model and its influence on IR, androgen levels, and the hypothalamic–pituitary–gonadal axis in a letrozole (LET)-induced PCOS rat model. In parallel, we performed phytochemical profiling and in vitro antioxidant assays of LSEE, along with an evaluation of its liver toxicity potential, to comprehensively assess the extract’s therapeutic profile. To our knowledge, no previous studies have evaluated the impact of *L. salicaria* L. extract on both DM and PCOS models, nor its ability to modulate shared pathogenic features such as OS, inflammation, and IR.

## 2. Materials and Methods

### 2.1. Chemicals

The materials utilized in this study were obtained from the following locations: FineTest Biotech Inc. (Wuhan, China)—ELISA kits for NF-κB, IL-1β, 3-NT, 8-OHdG, Insulin, Testosterone, Estrogen, FSH, and LH. ABclonal Technology (Woburn, MA, USA) and MyBioSource (San Diego, CA, USA)—kits for NLRP3 inflammasome biomarkers (IL-18 and Gasdermin) Merck (Darmstadt, Germany) and Sigma-Aldrich (Munich, Germany)—Folin-Ciocâlteu reagent, 2,2-diphenyl-1-picrylhydrazyl (DPPH), sodium nitroprusside, sodium carbonate, aluminum chloride, sodium acetate, methanol, acetate buffer, Griess-Ilosvay nitrite reagent, phosphate-buffered saline, sulphanilic acid, 2,4,6-tri(2-pyridyl)-1,3,5-triazine (TPTZ), xylenol orange, ortho-dianisidine dihydrochloride (3,3′-dimethoxybenzidine), thiobarbituric acid, ferric chloride, ethylenediaminetetraacetic acid, hydrogen peroxide (H_2_O_2_), sodium dodecyl sulfate, 1,1,3,3-tetrahydroxypropane, vanadium(III) chloride (VCl_3_), 5,5′-dithio-bis 2-nitrobenzoic acid (DTNB), butylated hydroxytoluene, Trolox (6-hydroxy-2,5,7,8-tetramethylchroman-2-carboxylic acid), N-(1-Naphthyl) ethylenediamine dihydrochloride (NEDD), and HPLC-grade acetonitrile. Spinreact S.A./S.A.U. (Girona, Spain)—Assay kits for AST, ALT, total cholesterol (TC), triglycerides (TG), and glycemia. Sandoz (Basel, Switzerland)—Letrozole; Millipore (Burlington, MA, USA)—Ultrapure water (obtained using a Direct-Q UV system). The pure standards of ellagic, gallic acid, and luteolin were purchased from Sigma-Aldrich (Munich, Germany).

### 2.2. Plant Material Processing

The seeds of *Lythrum salicaria* L. used in this study come from the germplasm collection of the Center for Biodiversity and Conservation of the University of Agricultural Sciences and Veterinary Medicine Cluj-Napoca, Romania, and were taxonomically identified and authenticated identified by the head of the Center for Biodiversity and Conservation, Prof. Dr. Cătană Corina (C.C.). The seed collection is based on the most effective and environmentally safe control practices known from recent research and experience. The experimental plants were grown in Sălaj County, Lat. 47.09.19.9/Long. 23.25.18.1, Romania, and plant material was collected during the flowering stage in August 2022.

The above-ground parts of *L. salicaria* L. were dried in a shaded, well-ventilated area at room temperature (25 ± 2 °C) with relative humidity maintained below 50%. The drying process lasted for 10 days, ensuring minimal exposure to direct sunlight and preserving thermolabile compounds.

The dried aerial parts of *L. salicaria* L. (300 g) were ground into a fine powder in a coffee grinder (Argis, RC-21, Electroarges SA, Curtea de Arges, Romania) for 5 min. Further, the plant material was subjected to extraction in the Mycology Laboratory of Babes-Bolyai University, Cluj-Napoca, Romania, using a modified Squibb percolation method. Briefly, plant material was loaded into three percolators as follows: 150 g in the first one, 90 g in the second one, and 60 g in the third one. Then, plant material was soaked with 150 mL 70% ethanol, and after two days, the three percolated fractions (60 mL, 90 mL and 120 mL) were collected and mixed. The resulting final extract had a concentration of 1:1 g/mL (*w*:*v*) in 30% ethanol, meaning 1 g of dry plant material (d.w.) was used per 1 mL of extract. This extract was then diluted with 0.9% saline solution to prepare the dosing solutions, resulting in a concentration of 100 mg d.w./mL (LSEE 100%). The tested concentrations were LSEE 100%, LSEE 50%, and LSEE 25%. LSEE 50% and LSEE 25% were obtained by diluting the LSEE 100% solution with a 0.9% saline solution in a 1:2 (50%) and 1:4 (25%) ratio, respectively, yielding final concentrations equivalent to 50 mg/mL and 25 mg/mL of dry plant material. All solutions were freshly prepared before administration.

### 2.3. Total Phenolic and Total Flavonoid Content Determination

The total phenolic content (TPC) of the *Lythrum salicaria* L. ethanol extract (LSEE) was determined using the Folin–Ciocalteu method, with gallic acid as the reference standard. Briefly, 2 mL of LSEE was mixed with 1 mL of Folin–Ciocalteu reagent, 10 mL of distilled water, and then diluted to 25 mL with a sodium carbonate solution (290 g/L). The resulting solution was incubated in the dark at room temperature for 30 min, after which the absorbance was measured at 760 nm using a JASCO V-530 UV-VIS spectrophotometer (Jasco, Tokyo, Japan). TPC was expressed as milligrams of gallic acid equivalents per gram of dry weight (mg GAE/g d.w.), based on a gallic acid calibration curve (R^2^ = 0.999) [2].

Total flavonoid content (TFC) was measured using an aluminum chloride method, as previously described [12]. Sodium acetate and aluminum chloride were added to the extract and diluted, and absorbance was recorded at 430 nm. TFC was expressed as mg quercetin equivalents (QE) per gram d.w., using a quercetin calibration curve (R^2^ = 0.999). Both determinations were performed in triplicate [3].

### 2.4. HPLC-DAD-ESI MS

An Agilent 1200 HPLC system (Agilent Technologies, Santa Clara, CA, USA), equipped with a quaternary pump, solvent degasser, autosampler, UV–vis diode-array detector (DAD), and coupled to a single quadrupole mass spectrometer (Agilent 6110), was employed for compound analysis. Separation was performed on a Kinetex XB C18 column (4.6 × 150 mm, 5 μm; Phenomenex, Torrance, CA, USA). The mobile phase consisted of (A) water with 0.1% acetic acid and (B) acetonitrile with 0.1% acetic acid, applied at a flow rate of 0.5 mL/min and a column temperature of 25 °C, using the following gradient (in % B): 0 min, 5%; 0–2 min, 5%; 2–18 min, 5–40%; 18–20 min, 40–90%; 20–24 min, 90%; 24–25 min, 90–5%; and 25–30 min, 5%. UV spectra were recorded over 200–600 nm, with chromatograms monitored at 280 and 340 nm.

Mass spectrometry (MS) was performed in full-scan positive ESI mode under the following conditions: capillary voltage, 3000 V; source temperature, 350 °C; nitrogen flow, 7 L/min; and scan range, *m/z* 120–1200. Data acquisition and processing were conducted using Agilent ChemStation software (Rev B.04.02 SP1). Compound identification was based on compounds retention times, their UV-Vis spectra, their mass spectra, and existing literature data.

Quantification of phenolic compounds was carried out using calibration curves from three concentrations of standards dissolved in methanol—ellagic acid (R^2^ = 0.9981), LOD = 0.51 μg/mL, LOQ = 1.53 μg/mL; gallic acid (R^2^ = 0.9978), LOD = 0.35 μg/mL, LOQ = 1.05 μg/mL, and luteolin (R^2^ = 0.9972), LOD = 0.18 μg/mL, LOQ = 0.54 μg/mL. The equations derived from these curves were used for the quantitative determination of each phenolic compound.

### 2.5. Evaluation of In Vitro Antioxidant Effects

Antioxidant activity was determined using the following in vitro assays: 2,2-diphenyl-1-picrylhydrazyl (DPPH) radical scavenging, ferric-reducing antioxidant power (FRAP), nitric oxide (NO) scavenging, and hydrogen peroxide (H_2_O_2_) scavenging assay. All in vitro assays were performed in triplicate.

DPPH Radical Scavenging Assay: The DPPH assay involved mixing equal volumes of sample solution with 0.1 mg/mL DPPH in methanol, followed by a 30 min dark incubation at room temperature. Absorbance at 517 nm was measured. The antioxidant capacity was quantified as IC_50_ values (μg/mL) and converted to TX equivalents (TE, μg TE/mL) using a calibration curve. Based on the TE values, antioxidant potential was classified as very good (<50 μg TE/mL), good (50–100 μg TE/mL), weak (100–200 μg TE/mL), or no significant potential (>200 μg TE/mL) [4,5].

Ferric-reducing antioxidant power (FRAP) Assay: The FRAP assay measured antioxidant activity by assessing the reduction of Fe^3+^ to Fe^2+^ using TPTZ. A reagent mixture of acetate buffer, TPTZ, and ferric chloride (10:1:1 *v*/*v*/*v*) was incubated for 30 min, and absorbance was read at 593 nm. Results were expressed as mg TX equivalents (TE) per mL [6].

Nitric Oxide (NO) Scavenging Assay: NO scavenging activity was determined using the Griess reagent method. LSEE was incubated with sodium nitroprusside (SNP) and phosphate-buffered saline (PBS) for 2.5 h. The resulting mixture was then reacted with sulphanilic acid and N-(1-Naphthyl) ethylenediamine dihydrochloride (NEDD). After a final 30 min incubation, absorbance was measured at 546 nm, and results were expressed as IC_50_ values (mg TE/mL) [7].

Hydrogen Peroxide (H_2_O_2_) Scavenging Assay: H_2_O_2_ scavenging activity was assessed by adding LSEE in water to a hydrogen peroxide solution. After 10 min, absorbance was measured at 230 nm. The percentage of H_2_O_2_ scavenged was calculated, and results were expressed as IC_50_ values (mg TE/mL of ethanolic plant extract) [8].

### 2.6. Experimental Design

The study protocol received approval from the Institutional Animal Ethical Committee (IAEC) of “Iuliu Hațieganu” University of Medicine and Pharmacy Cluj-Napoca, as well as authorization from the National Sanitary Veterinary and Food Safety Agency (approval no. 302/4 April 2022).

The study utilized adult female Wistar albino rats, each weighing between 200 and 250 g. They were housed in the Animal Facility of “Iuliu Hațieganu” University of Medicine and Pharmacy, maintained under controlled conditions—12 h light/dark cycle, temperature set at 22–24 °C, and humidity at 55–60%. Standard pelleted food (Cantacuzino Institute, Bucharest) and water were provided ad libitum.

For the experimental STZ-induced DM model, animals were distributed into 10 groups (*n* = 8):

- CONTROL: negative control group.
- STZ: positive control group with DM induced with STZ (55 mg/kg b.w.) [9].
- STZ + TX: DM receiving TX (20 mg/kg b.w.) [10].
- STZ + M: DM receiving Metformin (100 mg/100 g b.w.) [11].
- STZ + LSEE 100%: DM receiving LSEE 100% (0.5 mL extract/100 g b.w./day)
- STZ + LSEE 50%: DM receiving LSEE 50% (0.5 mL extract/100 g b.w./day)
- STZ + LSEE 50%: DM receiving LSEE 50% (0.5 mL extract/100 g b.w./day)
- STZ + LSEE 25%: DM receiving LSEE 25% (0.5 mL extract/100 g b.w./day)

The DM groups received the LSEE treatments by oral gavage, corresponding to the following doses: 50 mg/100 g b.w. (LSEE 100%), 25 mg/100 g b.w. (LSEE 50%), and 12.5 mg/100 g b.w. (LSEE 25%).

DM was induced on the first day, after an overnight fast, by a single STZ intraperitoneal injection (55 mg/kg) dissolved in 0.1 M citrate buffer (pH 4.5). Rats in the CONTROL group were injected with citrate buffer (pH 4.5) alone. Over a ten-day period, beginning from day one, CONTROL and STZ groups were given 1 mL/day tap water via gavage, whereas the LSEE groups received three concentrations of LSEE (100%, 50% and 25%). Starting on day two, fasting blood glucose was measured daily with an Accu-Check Active glucometer, followed by subcutaneous administration of 2–3 IU Insulin Aspart as necessary.

For the experimental LET-induced PCOS model, animals were divided into five groups (*n* = 8):

- CONTROL: negative control group;
- LET: positive control group with PCOS induced with LET (1 mg/kg b.w.) [12];
- LET + TX: PCOS receiving TX (20 mg/kg b.w., gavage);
- LET + M: PCOS receiving Metformin (100 mg/100 g b.w., gavage);
- LET + LSEE 100%: PCOS receiving LSEE 100% (0.5 mL extract/100 g b.w./day).

The PCOS group received the LSEE treatment by oral gavage at a dose of 50 mg/100 g b.w./day.

The treatments were administered starting on the 10th day of the experiment. In order to induce the PCOS phenotype, rats in LET groups were orally administered 1 mg/kg of LET dissolved in 0.5% Carboxy Methyl Cellulose (CMC) over a 21-day period, once daily. On day 10 of the experiment, an ultrasound examination was performed in order to confirm the presence of the polycystic ovaries.

On day 11 (DM rats) and day 22 (PCOS rats), blood samples were collected via retro-orbital puncture. General anesthesia was induced using a combination of ketamine (70 mg/kg) and xylazine (10 mg/kg). Serum samples were separated and preserved at −80 °C until further examination. For PCOS rats, ovaries were removed and fixed in 10% phosphate-buffered formalin (pH 7.0) for 24 h prior to histopathological examination. Ovarian weight and diameter were measured. At the study’s completion, all animals were euthanized by cervical dislocation.

### 2.7. Pharmacological Studies

#### 2.7.1. Serum Oxidative Stress Markers Evaluation

Total oxidative status (TOS) was measured using a colorimetric assay, which quantifies oxidants by converting ferrous ions (Fe^2+^) to ferric ions (Fe^3+^) in an acidic environment, detected using an xylenol orange reaction. An automated analyzer, calibrated with hydrogen peroxide, was used, and results were expressed in μM H_2_O_2_ equivalents/L [6].

Serum total antioxidant capacity (TAC), reflecting overall antioxidant defenses, was determined using a colorimetric method. The assay measured the capacity to neutralize dianisidyl radicals, which are generated by the oxidation of ortho-dianisidine. A standard solution of Fe^2+^-o-dianisidine undergoes a Fenton reaction with hydrogen peroxide, producing hydroxyl radicals. Antioxidants in the sample inhibit the oxidation of o-dianisidine and, thus, reduce color development. The color intensity was measured spectrophotometrically, and the assay was calibrated using TX. TAC values were expressed as mM TX equivalents (TE)/L [13].

The oxidative stress index (OSI), representing the balance between oxidants and antioxidants, was determined by dividing the TOS value (mM H_2_O_2_ equivalents/L) by the TAC value (mM TX equivalents/L) and expressed as an arbitrary unit [14].

Nitric Oxide (NO) levels were indirectly assessed by quantifying its stable end products, nitrite and nitrate (NOx), using the Griess reaction. Prior to the Griess reaction, nitrates were reduced to nitrites using Vanadium (III) chloride. Serum NOx concentrations were then determined spectrophotometrically and expressed as μM nitrites/L [15].

Serum malondialdehyde (MDA) concentration, an indicator of lipid peroxidation, was quantified using a thiobarbituric acid assay. Serum (150 μL) was mixed with trichloroacetic acid (125 μL of 10%), EDTA (125 μL of 5 mM), sodium dodecyl sulfate (125 μL of 8%), and butylated hydroxytoluene (10 μL of 0.5%). After incubation and centrifugation, the supernatant was reacted with thiobarbituric acid (500 μL of 0.6%) at 95 °C. Absorbance was measured at 532 nm, and MDA concentrations were determined using a 1,1,3,3-tetrahydroxypropane standard curve (0.3–10 nM/mL), with results expressed as nM/mL serum [16].

Serum concentrations of 8-Hydroxydeoxyguanosine (8-OHdG) and 3-Nitrotyrosine (3NT) were determined using commercially available ELISA kits according to the manufacturers’ instructions. Results were expressed in ng/mL.

Serum advanced glycation end product (AGE) concentrations were determined using a commercial ELISA kit. The results were expressed in U/mL.

Advanced oxidation protein products (AOPP) in serum were measured using a method adapted from Witko-Sarsat et al. Serum samples were diluted in PBS and reacted with glacial acetic acid and potassium iodide. Absorbance was measured at 340 nm. AOPP concentrations were expressed as µM chloramine-T equivalents/L [17].

Serum total thiol (SH) concentrations were quantified using Ellman’s reagent. The reagent reacts with thiol groups, and the resulting supernatant absorbance was measured spectrophotometrically at 412 nm. SH concentrations were expressed as mM reduced glutathione (GSH)/mL [18].

#### 2.7.2. Serum Inflammatory Markers Evaluation

Serum levels of NLRP3 inflammasome activation biomarkers (IL-1β, IL-18, NF-κB p65, and Gasdermin D) were quantified using ELISA kits according to the manufacturers’ instructions. IL-18, IL-1β, and NF-κB p65 concentrations were expressed in pg/mL. Gasdermin D levels were expressed in ng/mL.

#### 2.7.3. Blood Glucose, Lipid Profiles, Triglyceride-Glucose Index, Liver-Injury Makers, and Anthropometric Parameters Evaluation

To confirm successful hyperglycemia induction by STZ, glucose levels were initially assessed 24 h after STZ administration. Blood samples were taken from the dorsal tail vein, and glucose was measured using an Accu-Check Active glucometer with enzymatic strips. Subsequently, at the time of sacrifice (day 11), blood glucose levels were determined from retro-orbitally collected samples using commercial assay kits. Results were expressed in mg/dL.

Serum total cholesterol (TC) and triglyceride (TG) levels were quantified spectrophotometrically using commercial assay kits following the manufacturer’s instructions, with results expressed in mg/dL.

Triglyceride-glucose index (TyG), a marker of IR, was calculated using the formula TyG = Ln [fasting triglycerides (mg/dL) × fasting glucose (mg/dL)/2] at the end of the study [19].

Liver injury was investigated by measuring serum alanine aminotransferase (ALT) and aspartate aminotransferase (AST) levels using commercial assay kits. Results were expressed in U/L, and the AST/ALT ratio was calculated.

Body weight (BW) measurements were taken at the initiation of the study (day 1) and at the final time points (on days 11 and 22, respectively), and the change in body weight was calculated and expressed in grams (g).

#### 2.7.4. Hormonal Assays

Serum concentrations of insulin, follicle-stimulating hormone (FSH), luteinizing hormone (LH), estrogen, and testosterone were determined by ELISA using commercially available kits from Finetest (Wuhan, China). The assays were performed following the manufacturer’s instructions, and absorbance was measured at 450 nm ± 2 nm using a microplate reader.

The Homeostatic Model Assessment of IR (HOMA-IR) was calculated using the formula: [serum glucose (mg/dL) × serum insulin (mIU/mL)]/405.

### 2.8. Histopathological Assessment of Reproductive Organs

Following ethical guidelines and approved protocols, the animals were humanely euthanized. The ovaries were dissected out, defatted, and cleaned in saline. The weight of the ovaries and ovarian diameter were recorded. The ovaries were then subjected to fixation by immersion in 10% phosphate-buffered formalin (pH 7.0) for a period of 24 h. Standard histological procedures for paraffin embedding were employed, and sections of 3 µm thickness were generated using a microtome. Hematoxylin and eosin (H&E) staining was subsequently performed, and sections were analyzed using an Olympus BX-51 light microscope (Microscope Central, Willow Grove, PA, USA). Photomicrographs were captured with an Olympus SC 180 digital camera and processed using cellSense software v4.2.1 (Olympus Corporation, Tokyo, Japan) [20].

### 2.9. Ultrasound Examination

Ultrasound evaluations were performed using an Esaote MyLab 40 Vet system equipped with a linear probe transducer, with a frequency of 18 MHz. Anesthesia was induced with intraperitoneal administration of Xylazine (5 mg/kg) and Ketamine (60 mg/kg) [21]. The animals were shaved bilaterally from the costal margin to the caudal abdomen, and a generous amount of ultrasound gel was applied. Rats were positioned in dorsal recumbency. Ultrasound was performed on day 10 of the experiment by two operators, with both present at each evaluation. The transducer was moved along the vertical axis and horizontal axis (forward-to-back and side-to-side) by hand. The ovarian size along with the appearance and size of the follicles were observed.

### 2.10. Statistical Analysis

SPSS v26.0 (SPSS Inc., Chicago, IL, USA) and Statistica 12 (TIBCO Software, Palo Alto, CA, USA) software packages were used for all statistical computations. For normally distributed data, results are presented as mean ± standard deviation (SD). To determine significant differences between groups, a one-way ANOVA was conducted, followed by Bonferroni–Holm post hoc tests for pairwise comparisons. Pearson’s correlation analysis was used to assess correlations between biomarkers. Statistical significance was set at *p* < 0.05. Principal Component Analysis (PCA) was utilized for multivariate analysis of the datasets. Bartlett’s test was used to assess the suitability of the data for factor analysis, and the Kaiser–Meyer–Olkin (KMO) criterion was used to evaluate sampling adequacy.

## 3. Results

### 3.1. Phytochemical Analysis

**Figure 1:**
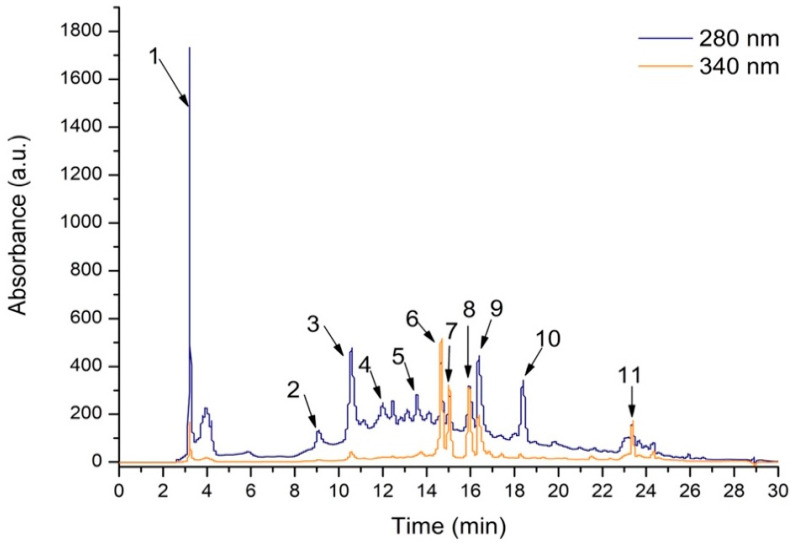
The HPLC chromatogram of phenolic content from LSEE at 340 nm.

In LSEE, the TPC was quantified at 183.9 mg GAE/100 g of dry weight plant material, whereas the TFC reached 8.34 mg QE/100 g d.w. of plant material. HPLC-DAD-ESI MS identified significant concentrations of flavones, hydroxybenzoic acids, and ellagitannins. In total, 11 compounds were identified, from which 4 compounds were flavones, 4 compounds were hydroxybenzoic acids, 2 compounds were ellagitannins, and 1 compound belonged to the gallotanin subclass of phenolic compounds. Among flavone compounds, the luteolin derivatives were the most represented ones. Among hydroxybenzoic acids, the ellagic acid derivatives were the most represented ones (Figure 1, Table 1).

The LSEE contains four hydroxybenzoic acids: 2-Hydroxybenzoic acid corresponding to peak 1, with *m/z* 139, and three ellagic acid derivatives corresponding to peak 5, 9, and 10, with *m/z* 465, 303, and 792. Four flavonoid derivatives were identified: Luteolin-galactoside corresponding to peak 6, with *m/z* 449, Luteolin-glucoside corresponding to peak 7 with *m/z* 449, Apigenin-glucoside corresponding to peak 8 with *m/z* 433 and luteolin corresponding to peak 11 with *m/z* 287. From the ellagitannin derivatives, Castalagin corresponding to peak 2, with *m/z* 935 and Vescalagin corresponding ot peak 3, with *m/z* 935, were identified. From the gallotanin derivatives, the identified compound was Digalloyl-glucoside corresponding to peak 4, with *m/z* 485 (Table 1)**.**

### 3.2. The In Vitro Oxidative Stress Markers

LSEE exhibited a good in vitro antioxidant activity with all the tests. DPPH assay showed a good antioxidant activity, with the result being between 50 and 100 μg TE/mL (*p* < 0.001). LSEE H_2_O_2_ scavenging activity (*p* < 0.001) and FRAP (*p* < 0.001) tests were better as compared to TX. LSEE NO scavenging activity was also better as compared to quercetin (*p* < 0.01) (Table 2).

### 3.3. Confirmation of Rodent DM and PCOS Models

The presence of STZ-induced DM was confirmed by measuring blood glucose levels of at least 250 mg/dL on the second day of the experiment. All rodents administered STZ developed significant hyperglycemia by day two and were subsequently included in the study.

The induction of PCOS in rats was confirmed after 21 days of LET administration, as indicated by significantly elevated gonadotropins (*p* < 0.01), reduced estrogen (*p* < 0.001), and increased testosterone levels (*p* < 0.05) when compared to the CONTROL. Furthermore, metabolic disturbances were observed, including significantly elevated insulin levels (*p* < 0.001), as well as an increased HOMA-IR index (*p* < 0.001), indicating the presence of IR. Ultrasound imaging revealed polycystic ovarian morphology, confirming the successful induction of PCOS. Prominent histological changes were also described in the ovaries of LET-treated rats. In the LET group, the ovaries contained numerous large cystic structures, and there was a marked absence or reduced presence of corpus luteum.

### 3.4. The In Vivo Oxidative Stress Markers

**Figure 2:**
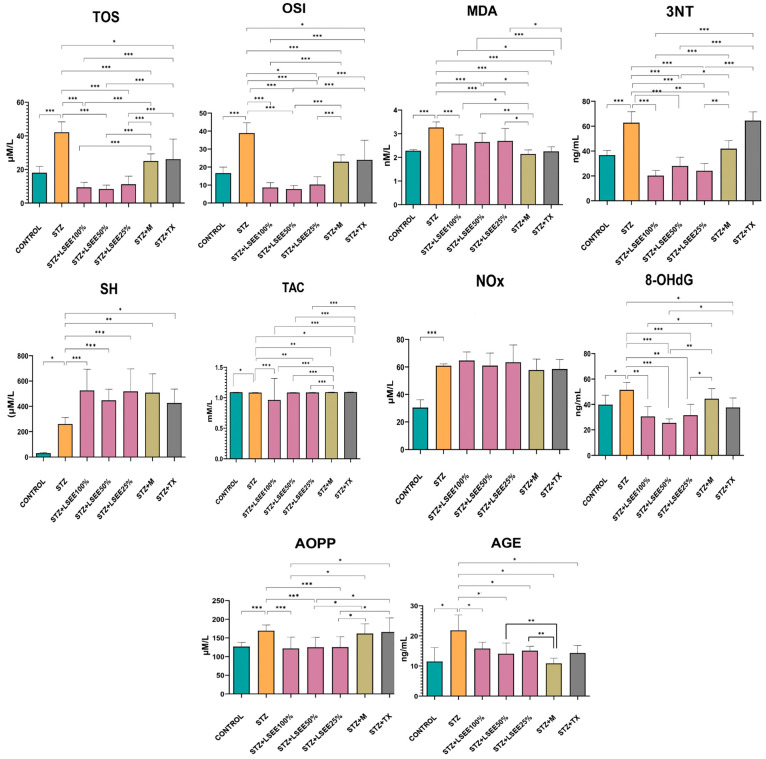
Effects of *L. salicaria* on oxidative stress markers in rat STZ-induced DM vs. CONTROL: * *p* < 0.05; ** *p* < 0.01, *** *p* < 0.001; STZ—Streptozotocin; STZ + TX—STZ + Trolox; STZ + M—STZ + Metformin; LSEE—*L. salicaria* L. ethanol extract; TOS—Total oxidative status; TAC—Total antioxidant capacity; OSI—Oxidative stress index; MDA: Malondialdehyde; AOPP—Advanced oxidation protein products; 8-OHdG—8-hydroxydeoxyguanosine; NOx—Nitrites and nitrates; 3NT—3-nitrotyrosine; AGEs—Advanced glycation end product; SH—total thiols.

When comparing the oxidative stress parameters in the STZ to the CONTROL there were significant increases in the oxidants TOS, OSI, MDA, Nox, and AOPP (*p* < 0.001), 3NT (*p* < 0.01), and 8-OHdG and AGEs (*p* < 0.05). The antioxidant defense markers TAC and SH were significantly decreased (*p* < 0.05) (Figure 2).

By comparing the LSEE effects with the STZ group, we found that TOS, OSI, MDA, AOPP, and 3NT were significantly reduced by all of the three concentrations of LSEE (*p* < 0.001). 8-OHdG was significantly lowered by LSEE 50% (*p* < 0.001), LSEE 100%, and LSEE 25%, causing a milder but also significant reduction (*p* < 0.01). AGEs were reduced by all of the LSEE treatments in the same manner (*p* < 0.05). LSEE exhibited no effect on NOx. The extract improved antioxidant defense by increasing SH, significantly for all of the three concentrations (*p* < 0.001). LSEE 100% significantly increased TAC (*p* < 0.01), while LSEE 25% caused a mild increase in TAC (*p* < 0.05) (Figure 2).

Metformin treatment managed to significantly reduce TOS, OSI and MDA (*p* < 0.001). At the same time, metformin significantly increased TAC and SH (*p* < 0.01), but LSEE in all concentrations caused a better increase in TAC (*p* < 0.001). AGEs and 3NT were also significantly reduced by metformin (*p* < 0.01). By comparing LSEE effects with STZ + M we found a significant difference in TOS and OSI (*p* < 0.001), LSEE causing a better reduction in all concentrations. LSEE caused better reduction of MDA, AOPP, 8-OHdG, and 3NT in all concentrations when compared to metformin (Figure 2). AGEs were better lowered by metformin, and the difference was statistically significant when compared to LSEE in all of the three concentrations (*p* < 0.01).

The treatment with TX reduced TOS, OSI, 8-OHdG and AGEs (*p* < 0.05) but had no effect on AOPP, NOx and 3NT (*p* > 0.05). TX increased TAC and SH levels (*p* < 0.05). By comparing TX effects with LSEE, we found significant differences in the reduction of TOS, OSI, and 3NT (*p* < 0.001) by LSEE in all concentrations. (Figure 2). MDA and AOPP were also significantly decreased by LSEE when compared to TX (*p* < 0.05), LSEE50% causing a better reduction of 8-OHdG (*p* < 0.01). LSEE in all of the three concentrations caused a better increase in TAC (*p* < 0.001) when compared to TX.

**Figure 3:**
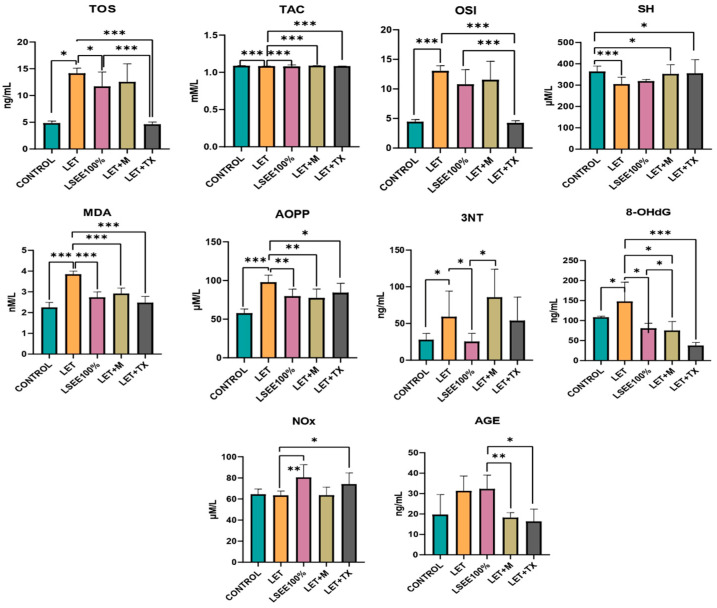
Effects of *L. salicaria* on oxidative stress markers in rat Letrozole-induced PCOS. vs. CONTROL: * *p* < 0.05, ** *p* < 0.01, *** *p* < 0.001; LET—Letrozole; LET + TX—Letrozole + Trolox; LET + M—Letrozole + Metformin; LSEE—*L. salicaria* L. ethanol extract; TOS—Total oxidative status; TAC—Total antioxidant capacity; OSI—Oxidative stress index; MDA: Malonyldialdehide; AOPP—Advanced oxidation protein products; 8-OHdG—8-hydroxydeoxyguanosine; NOx—Nitrites and nitrates; 3NT—3-nitrotyrosine; AGEs—Advanced glycation end product; SH—total thiols.

When comparing the oxidative stress parameters in the LET to the CONTROL there were significant increases in the oxidants TOS, OSI, MDA, and AOPP (*p* < 0.001) and 8-OHdG and 3NT (*p* < 0.05). The antioxidant defense markers TAC and SH were significantly decreased (*p* < 0.001) (Figure 3).

By comparing the LSEE100% effects with the LET group, we found that MDA was significantly reduced by LSEE100% (*p* < 0.001). LSEE 100% caused a reduction in AOPP (*p* < 0.01), TOS, 8-OHdG, and 3NT (*p* < 0.05). LSEE100% had no effect on AGEs, and it increased NOx levels. The extract improved antioxidant defense by increasing TAC (*p* < 0.001) (Figure 3).

The treatment with metformin reduced significantly MDA (*p* < 0.001), AOPP (*p* < 0.01) and 8-OHdG. At the same time, metformin significantly increased TAC (*p* < 0.001). By comparing LSEE effects with SZT + M, we found a significant reduction of 3NT by LSEE100% (*p* < 0.05). Metformin caused a better reduction of AGEs when compared to LSEE100% (*p* < 0.01) (Figure 3).

The treatment with TX reduced TOS, OSI (*p* < 0.05), MDA, 8-OHdG (*p* < 0.001), AOPP, and NOx (*p* < 0.05). TX increased TAC (*p* < 0.001) and SH (*p* < 0.05). By comparing TX effects with LSEE, we found a significant reduction of TOS and OSI (*p* < 0.001), respectively, of 8-OHdG (*p* < 0.01) and AGEs (*p* < 0.05) (Figure 3).

### 3.5. The In Vivo Inflammatory Markers

NLRP3 inflammasome activation, indicated by IL-1β, IL-18, NFκB-p65, and Gasdermin D levels, was used to assess the inflammatory response. Comparing the levels of inflammatory markers between the STZ group and the CONTROL group, we found a statistically significant increase in IL-1β and NFkB-p65 (*p* < 0.001), respectively, of Gasdermin D and IL-18 (*p* < 0.01) in the STZ group. LSEE in all three concentrations managed to reduce Gasdermin, IL-1β, and IL-18 levels (*p* < 0.001) significantly (Table 3). NFkB-p65 was significantly reduced only by LSEE 50% (*p* < 0.001) and LSEE 25% (*p* < 0.01). Caspase-1 was significantly reduced by LSEE50% (*p* < 0.05) and LSEE 25% (*p* < 0.001).

IL-1β (*p* < 0.001) and NFkB-p65 and IL-18 (*p* < 0.01) were significantly decreased by metformin treatment. LSEE at all three concentrations had a significantly stronger inhibitory effect than metformin on Gasdermin D and IL-18 (*p* < 0.001). LSEE 50% (*p* < 0.05) and LSEE 25% (*p* < 0.01) caused a better reduction of NFkB-p65 when compared to metformin. IL-1β was significantly reduced by metformin when compared to LSEE 100% and 20% (*p* < 0.01) and LSEE 50% (*p* < 0.05) (Table 3).

Upon comparing inflammatory markers between the TX and STZ groups, we determined a significant reduction in IL-1β and IL-18 (*p* < 0.001), NFkB-p65 (*p* < 0.01), and Gasdermin D (*p* < 0.05). By comparing TX effects with LSEE, we found significant differences in the reduction in inflammatory markers (Table 3).

**Figure 4:**
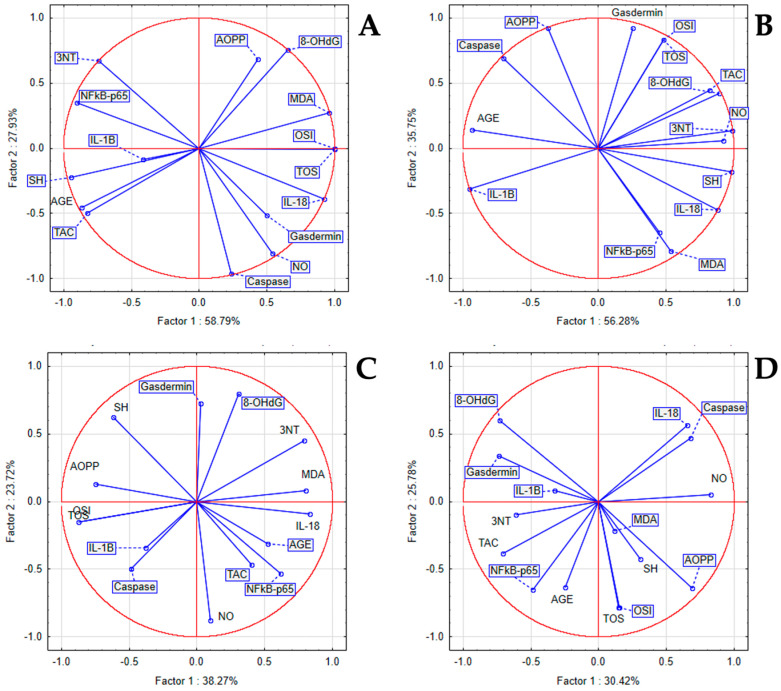
Oxidative stress and inflammatory markers PCA results: (**A**) STZ group; (**B**) LSEE 100% group; (**C**) LSEE 50% group; and (**D**) LSEE 25% group.

Within the STZ group, we found a statistically significant positive correlation between inflammatory and oxidant marker levels, and statistically significant negative correlations with antioxidant markers. PCA displayed a strong positive correlation between IL-18 with TOS, OSI, and MDA, and a milder correlation to 8-OHdG and AOPP. Within the LSEE 100% group, associations were found between IL-18 and NFκB-p65 levels and markers such as NO, MDA, SH, 8-OHdG, AOPP, and 3NT. Additionally, Gasdermin levels showed significant relationships with TOS, OSI, and TAC, whereas Caspase displayed a robust association with AGEs. In the LSEE 100% group, IL-18 and NFkB-p65 were correlated with MDA, NO, SH, AOPP, 8-OHdG, and 3NT, while Gasdermin was correlated with TOS, OSI, and TAC, and Caspase was strongly correlated with AGEs. In the LSEE 50% group, IL-18 was correlated to 3NT, MDA, and AGE, NFkB-p65 was correlated to AGE, TAC, and NO, while IL-1β and Caspase were correlated to TOS, OSI, and AOPP, and Gasdermin was correlated to 8-OHdG and SH. NfkB-p65 was correlated to AGE, TAC, and 3NT, and mildly correlated to TOS, OSI, MDA, AOPP, and SH, while Caspase and IL-18 were correlated to NO, and Gasdermin with 8-OHdG in the LSEE 25% group (Figure 4).

When comparing the inflammatory response obtained in the LET group to CONTROL, a statistically significant increase in NFkB-p65, IL-1β, and IL-18 (*p* < 0.01), and of anti-inflammatory cytokine IL-10 (*p* < 0.001) was observed. LSEE100% managed to significantly reduce NFkB-p65, IL-1β (*p* < 0.01), and IL-10 (*p* < 0.05) when compared to LET (Table 4).

IL-18 and IL-10 (*p* < 0.001) and IL-1β (*p* < 0.01) were significantly reduced in the metformin group. LSEE100% showed superior inhibitory activity compared to metformin, but only for NFκB-p65 and IL-10 (*p* < 0.01) (Table 4).

Comparing inflammatory markers between the TX and STZ groups, we found a significant reduction in NFkB-p65, IL-18, IL-10 (*p* < 0.05), and IL-1β (*p* < 0.01). We found significant differences in the reduction in inflammatory markers by comparing TX effects with LSEE 100% (Table 4).

**Figure 5:**
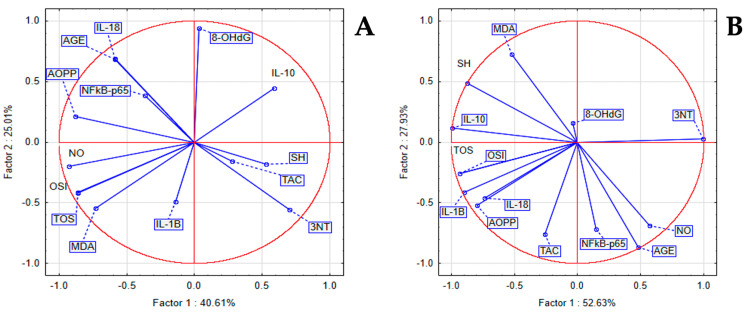
PCA results of oxidative stress and inflammatory markers in Letrozole induced PCOS in rats (**A**) LET group; (**B**) LSEE 100% group.

In the LET group, between inflammatory and oxidant markers, we noticed a statistically significant positive correlation and a significant negative correlation to antioxidant system parameters. PCA indicated that a positive correlation between NFkB-p65, IL-18 with AGEs, 8-OHdG, and AOPP was present. In the same group, IL-10 was correlated to TAC, SH, and 3NT, while IL-1β was correlated to TOS, OSI, MDA, and NO. In the LSEE 100% group, NFkB-p65 was correlated with AGE, NO and TAC, IL-18, and IL-1β were correlated to TOS, OSI, and AOPP, while IL-10 was correlated to SH, and mildly correlated to MDA and 8-OHdG (Figure 5).

### 3.6. The In Vivo Hypoglycemic, Lipid-Regulating, and Liver-Protective Effects

**Figure 6:**
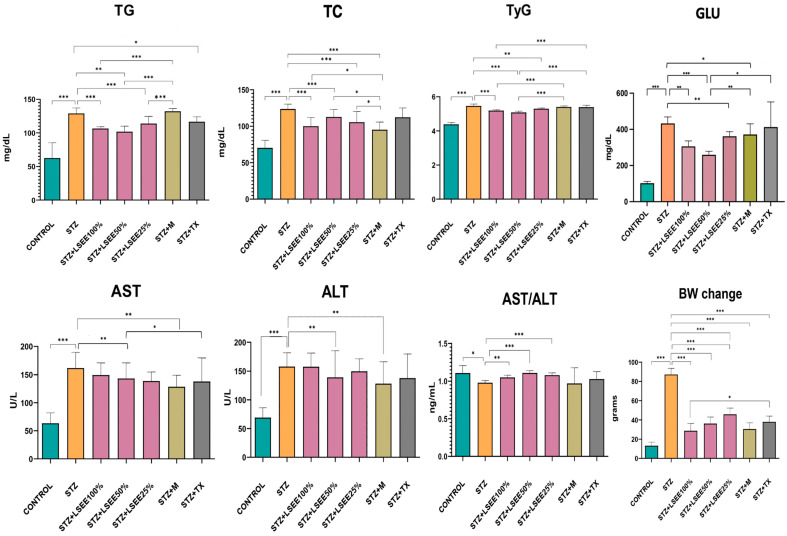
Markers indicative of glucose-lowering, lipid-regulating, and liver-protective activities in rat STZ-induced DM. vs. CONTROL: * *p* < 0.05, ** *p* < 0.0; *** *p* < 0.001; STZ—Streptozotocin; LSEE—*L. salicaria* L. ethanol extract; TX—Trolox; M—Metformin; AST—aspartate amino-transferase; ALT—alanine amino-transferase; AST/ALT—aspartate to alanine amino-transferase ratio; TC—total cholesterol; TG—triglyceride; GLU—glucose; TyG index—triglyceride to glucose index; BW—body weight.

Compared to the CONTROL group, the STZ group exhibited a significant increase in glucose (GLU) levels (*p* < 0.001), confirming diabetes mellitus (DM). Only animals with GLU levels of 250 mg/dL or higher were included. LSEE 50% reduced GLU (*p* < 0.001) and the effect was better than that of metformin (*p* < 0.01). LSEE 100% and LSEE 25% caused a smaller reduction in GLU (*p* < 0.01), but the effect of LSEE 100% was better when compared to metformin (*p* < 0.05) (Figure 6).

The evaluation of lipid metabolism profiles indicated a significant increase in TC and TG in the STZ groups compared to CONTROL (*p* < 0.001). Tyg index was also significantly increased in STZ animals (*p* < 0.01). TC and TG was reduced by all of the three concentrations (*p* < 0.001) when compared to STZ. TyG was also reduced by all of the three concentrations in a different manner, LSEE 50% causing the most significant reduction (*p* < 0.001), followed by LSEE 100% (*p* < 0.01) and LSEE 25% (*p* < 0.05). Metformin treatment caused a significant reduction in TC (*p* < 0.001) but had no effect on TG. LSEE 50% and LSEE 25% caused a significant reduction in TC when compared to metformin (*p* < 0.05) (Figure 6).

In the STZ group liver injury was confirmed by increases in ALT and AST levels (*p* < 0.001) and decrease in the AST/ALT ratio (*p* < 0.05) when compared to CONTROL. LSEE 50% caused a significant reduction in AST and ALT (*p* < 0.05) and ALT (*p* < 0.01). The AST/ALT ratio was significantly increased by LSEE 50% and LSEE 25% (*p* < 0.001); LSEE 100% caused a milder increase (*p* < 0.01). Metformin significantly reduced AST and ALT (*p* < 0.001) when compared STZ but had no effect on the AST/ALT ratio. Compared to LSEE concentrations, metformin caused a better reduction in AST and ALT, but the difference was not statistically significant. TX administration did not exhibit any lowering effect on liver injury markers. LSEE, metformin, and TX did not significantly influence the AST/ALT ratio compared to the STZ (*p* > 0.05) (Figure 6).

STZ administration led to a marked reduction in BW, resulting in a significant decrease by the end of the experiment compared to the initial BW. When compared to the BW change in the CONTROL group, the difference was statistically significant (*p* < 0.001). Treatment with LSEE at all three concentrations significantly increased BW compared to the STZ group (*p* < 0.001), with effects similar to those observed in metformin and TX-treated groups (*p* < 0.001) (Figure 6).

**Figure 7:**
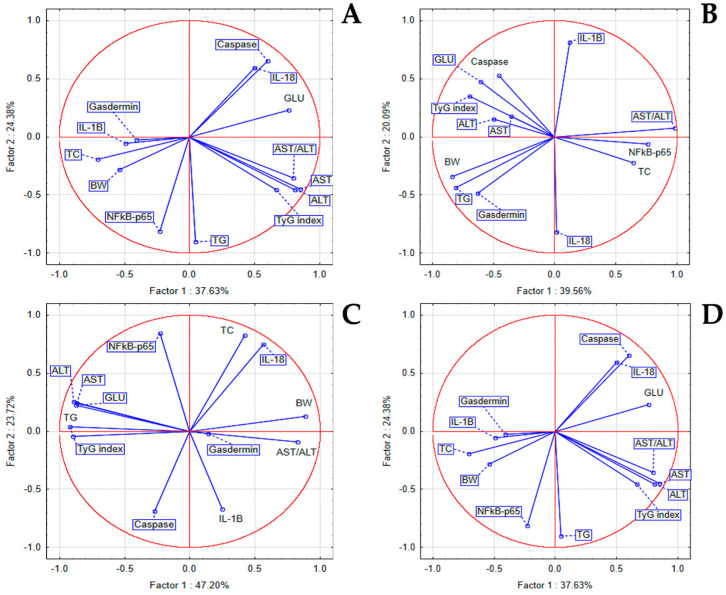
Oxidative stress, metabolic, and liver injury marker PCA results in STZ-induced DM in rats: (**A**) STZ group; (**B**) LSEE 100% group; (**C**) LSEE 50% group; and (**D**) LSEE 25%. STZ—STZ; STZ—STZ; LSEE—*L. salicaria* L. ethanol extract.

In the STZ group, PCA revealed correlations between NFκB-p65 and IL-1β with GLU, TG, and TyG index, and between IL-18 and TC, and a milder correlation between IL-1β and IL-18 to AST, ALT, and BW. In the LSEE 100% group, Gasdermin D was strongly correlated to TG and GLU, Caspase was correlated to GLU, TyG, AST, and ALT, while NFkB-p65 was correlated to TC and the AST/ALT ratio. In the LSEE 50% group, correlations were observed between NFκB-p65 and IL-1β levels and TC, while Gasdermin showed a strong association with the AST/ALT ratio and BW. In the same group, we noticed a weak correlation between NFkB-p65 and Caspase with AST, ALT, GLU, TyG index, and TG. In the LSEE 25% group, NF-κB p65 was correlated with TG, Il-1β, and Gasdermin with TC and BW, while IL-18 and Caspase were correlated with GLU (Figure 7).

### 3.7. Effects of L. salicaria L. Ethanolic Extract on Sex Hormones, Serum Insulin and HOMA-IR

When comparing the insulin levels in the LET group to the CONTROL we found a statistically significant increase in insulin (*p* < 0.001), GLU (*p* < 0.05) and HOMA-IR index (*p* < 0.001). LSEE 100% significantly reduced GLU (*p* < 0.01) and HOMA-IR (*p* < 0.001) but caused milder reduction in insulin levels with no statistical significance. Treatment with metformin and TX caused a significant reduction in insulin (*p* < 0.01), both having a stronger effect than LSEE100% (*p* < 0.001). GLU (*p* < 0.01) was significantly reduced by metformin administration, and the result was comparable to LSEE 100%. HOMA-IR was lowered by metformin and TX, and the reduction was significant when compared to LSEE 100% (*p* < 0.001) (Table 5).

Treatment with LET caused a significant increase in gonadotropins levels—FSH and LH (*p* < 0.01)—when compared to the CONTROL, while estrogen levels were lowered (*p* < 0.001), confirming the presence of the positive feedback on the pituitary hormones. Treatment with LSEE 100% significantly lowered LH (*p* < 0.01) and FSH (*p* < 0.05) and increased estrogen levels (*p* < 0.001). Metformin and TX managed to significantly reduce LH and estrogen levels (*p* < 0.001) but had no effect on FSH (Table 5).

Compared to the control group, testosterone levels were significantly elevated in the LET group (*p* < 0.05). LSEE 100% treatment significantly reduced testosterone levels in the LET group (*p* < 0.05), whereas metformin and TX had no significant effect (Table 5).

**Figure 8:**
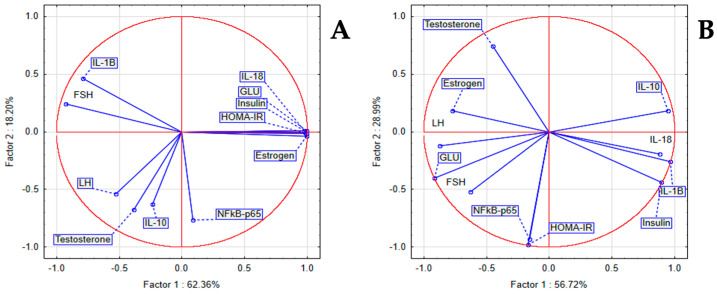
Hormonal and inflammatory markers PCA results in Letrozole induced PCOS in rats: (**A**) LET group; (**B**) LSEE 100% group. LSEE—*L. salicaria* L. ethanol extract.

In the LET group between inflammatory and hormonal markers, we noticed a statistically significant positive correlation between IL-18 with insulin, GLU, HOMA-IR, and estrogen. PCA indicated that a positive correlation between IL-1β and FSH was present, while IL-10 was correlated to LH and testosterone. In the LSEE 100% group, NFkB-p65 was correlated with HOMA-IR, and IL-18, IL-1β, and IL-10 were correlated with Insulin (Figure 8).

### 3.8. Effect of L. salicaria L. Ethanolic Extract on Ovarian Histology in Letrozole-Induced PCOS

**Figure 9:**
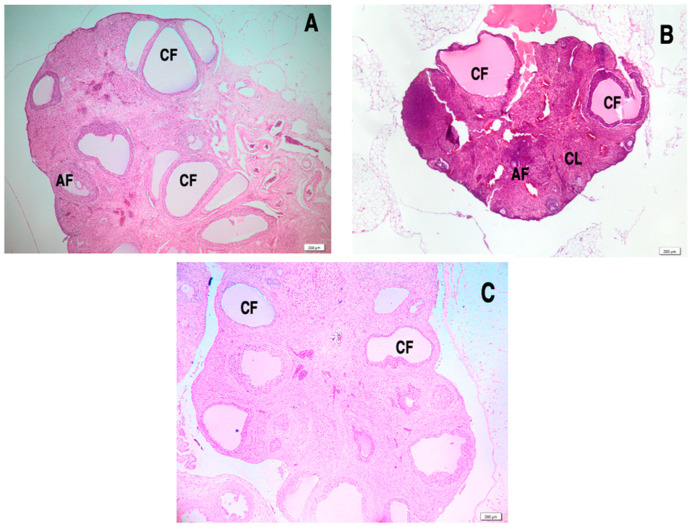
Effect of LSEE treatment on histology of letrozole-induced ovary. (**A**) LET group, (**B**) LET + LSEE 100% group, and (**C**) LET + M group. AF, Antral follicle; CL, corpus luteum; CF, cystic follicle.

H&E staining revealed significant histological changes in the ovaries of LET-treated rats. In the LET group, the ovaries displayed numerous large cystic structures, and an absence or scarcity of corpus luteum (Figure 9A). In these structures, the granulosa cells were sparse and arranged in two to three layers on the basal membrane, leading to the development of atretic follicles with a large central cavity. Notably, treatment with LSEE and metformin improved ovarian histology by reducing or eliminating cysts and decreasing the number of atretic antral follicles. Additionally, the previously aggregated and thickened intra-follicular granulosa cell layer of the large follicles showed signs of recovery, and corpus luteum formation was observed following LSEE and metformin treatment (Figure 9B,C).

### 3.9. Ultrasonography Examination

**Figure 10:**
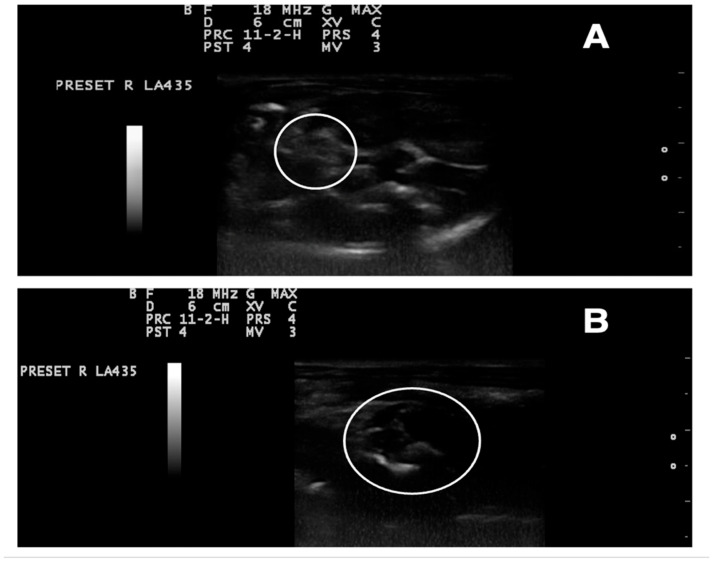
Ultrasonography analysis of ovary of PCOS animals. (**A**) CONTROL group: normal shape number of follicles (white circle). (**B**) LET group: enlarged, oblong, multiple cystic follicles (white circle).

The presence of ovarian cysts in the LET group was confirmed by ultrasonography examination on day 10 of the experiment. The USG findings showed that the ovaries of rats in this group were enlarged, with multiple oblong-shaped cystic structures, clearly indicating the development of cystic ovaries (Figure 10).

## 4. Discussion

Our study investigated the therapeutic potential of *Lythrum salicaria* L. ethanolic extract (LSEE) to address key pathophysiological mechanisms in both diabetes mellitus (DM) and polycystic ovary syndrome (PCOS), given the frequent association and shared underlying mechanisms between these two conditions. While current treatments for DM primarily manage glycemic control and are associated with side effects [22], naturally derived agents offer a promising avenue for therapeutic intervention [23,24]. Our findings demonstrated that LSEE significantly reduced blood glucose levels in STZ-induced diabetic rats, suggesting a potential antidiabetic effect. Furthermore, we also evaluated the impact of LSEE on hormonal and metabolic parameters in a LET-induced PCOS rat model, the results of which are discussed later in this section.

The Lythraceae family, to which *L. salicaria* L. belongs, includes several species with reported antidiabetic properties [24,25]. Although *L. salicaria* L. has shown promise for its anti-inflammatory and antioxidant activities, its antidiabetic efficacy has remained uncertain [26]. Our observation of significant blood glucose reduction in the STZ group contrasts with the mild hypoglycemic effect reported by Manayi et al. (2013) [22], who used a high dose (15 g/kg) of an aqueous methanol extract. This discrepancy in outcomes likely arises from variations in extraction methodologies, particularly the type of solvent employed. Furthermore, direct comparison with earlier studies like that of Lamela et al. (1986) [23], which suggested an antidiabetic potential, is challenging due to methodological differences and limited detailed data. Collectively, these findings, including our own, underscore the need for further standardized investigation to definitively establish the antidiabetic efficacy of *L. salicaria* L.

TPC and TFC of LSEE determined by Folin–Ciocâlteu method in our study were similar to other studies findings, confirming that *L. salicaria* L. are a significant source of phenolic compounds [22,24]. The most frequently identified phenolic compounds in LSEE belonged to the subclasses of hydroxybenzoic acids, with ellagic acid derivatives being the most abundant. Additionally, flavonoid subclasses, particularly luteolin and apigenin derivatives, as well as ellagitannin subclasses represented by casuarictin and vescalagin, were identified. Previous studies re ported that ellagitannins and ellagic acid derivatives were among the most frequently identified compounds in *L. salicaria* L. extracts [1,25]. Ellagitannins, particularly casuarictin, have been identified as potent inhibitors of enzymes such as α-glucosidase and α-amylase, which are crucial for carbohydrate digestion [26,27]. Vescalagin has also been reported to possess hypoglycemic, hypotriglyceridemic, and insulin-sensitizing properties [28]. Ellagic acid has been demonstrated to stimulate insulin secretion from pancreatic β-cells and to enhance insulin sensitivity, particularly in hepatic tissues [29,30]. As antioxidants, ellagic acid and ellagitannins scavenge ROS and RNS, reducing oxidative damage, enhancing the activity of antioxidant enzymes like SOD and catalase, and preventing lipid peroxidation [31,32]. As anti-inflammatory agents, they inhibit the NF-κB pathway, reducing pro-inflammatory cytokines such as TNF-α and IL-1β, and suppress COX-2 activity, which lowers prostaglandin synthesis [33,34]. Additionally, ellagic acid and ellagitannins regulate the NLRP3 inflammasome, decreasing inflammation [27]. The antidiabetic properties of luteolin and apigenin derivatives are mainly attributed to their ability to improve insulin sensitivity, by enhancing insulin signaling through activating key proteins such as AMP-activated protein kinase (AMPK) and improving the function of insulin receptors [35,36]. Additionally, luteolin, and apigenin derivatives have been demonstrated to regulate glucose homeostasis by inhibiting enzymes like α-glucosidase and α-amylase, thereby slowing down glucose absorption and preventing postprandial hyperglycemia [36,37]. Apigenin and luteolin exert their antioxidative and anti-inflammatory effects by scavenging free radicals, enhancing antioxidant enzyme activity, and inhibiting pro-inflammatory pathways such as NF-κB and MAPK signaling [38,39].

LSEE demonstrated strong in vitro antioxidant activity across multiple assays, suggesting its bioactive compounds contribute to its free radical scavenging potential. Its performance was either superior or comparable to standard references, with variations in activity attributed to the specific mechanisms each test evaluates. LSEE exhibited high DPPH scavenging activity, likely due to phenolic and flavonoid compounds (e.g., ellagic acid, luteolin, apigenin derivatives, and tannins), which are well-known for their electron-donating abilities. LSEE showed significantly higher hydrogen peroxide neutralization than TX, the superior performance of LSEE in this assay suggesting the presence of phenolic acids and flavonoids, which have been previously reported to act as hydrogen peroxide scavengers by acting as electron donors. LSEE exhibited significantly higher FRAP activity compared to TX, indicating a strong reducing power. This result suggests that LSEE contains potent electron-donating compounds capable of reducing metal ions and preventing oxidative stress induced by Fe^3+^-mediated reactions. LSEE also exhibited a superior nitric oxide scavenging compared to quercetin, likely due to ellagic acid derivatives and flavonoids, which help prevent NO-induced OS [40]. Our results were consistent with those reported in previous studies [41].

For the evaluation of in vivo effects of LSEE, the present study firstly used a rat STZ-induced T1DM model. STZ, a glucosamine-nitrosourea compound derived from *Streptomyces achromogenes*, is widely used to induce T1DM in rodent models. Its glucose analog structure facilitates selective entry into pancreatic beta cells via GLUT2 transporters, leading to DNA damage, reactive oxygen species (ROS) generation, and, ultimately, beta-cell destruction [42,43]. In this study, a single intraperitoneal STZ injection was employed to induce T1DM in rats. Successful induction was confirmed by significant hyperglycemia within 48 h, reflecting the rapid and severe beta-cell loss characteristic of this model.

In DM, chronic hyperglycemia leads to excessive production of ROS and reactive nitrogen species (RNS) through several interconnected pathways. Elevated glucose levels increase mitochondrial electron transport chain activity, leading to leakage of electrons and excessive ROS production. In hyperglycemic conditions, excess glucose is shunted into the polyol pathway, where aldose reductase converts glucose into sorbitol, consuming NADPH and depleting glutathione (GSH), a major antioxidant. This reduces the cell’s ability to counteract oxidative damage, leading to ROS overproduction. Chronic hyperglycemia causes non-enzymatic glycation of proteins, lipids, and DNA, forming advanced glycation end-products (AGEs), which interact with their receptors (RAGEs) to trigger oxidative stress and inflammatory responses. Another involved mechanism is represented by NADPH oxidase (NOX) activation, further amplifying ROS generation and contributing to vascular endothelial dysfunction [44,45]. Moreover, in DM, ROS can contribute to long-term dysfunctions that persist even after glycemic control is achieved, a phenomenon known as “metabolic memory” [46].

The findings of this study demonstrate that the STZ-induced T1DM model exhibited significant OS. Notably, OS biomarkers showed an inverse correlation with antioxidant system parameters, which were reduced when compared to the control group. Treatment with LSEE at all tested concentrations effectively reduced TOS and OSI, biomarkers that reflect the total concentration of oxidant molecules, while increasing TAC, an indicator of the body’s overall antioxidant capacity [47]. Furthermore, the antioxidant effect of metformin and TX, which also contribute to ROS reduction, was found to be lower than that of LSEE.

MDA serves as a key indicator of lipid peroxidation, reflecting oxidative damage to cell membranes. In LSEE-treated STZ rats, MDA levels were significantly reduced, suggesting that LSEE effectively mitigates ROS-induced lipid peroxidation. Similarly, AOPP, a biomarker of plasma protein oxidation, was also lowered by LSEE through the same ROS-reducing mechanism. This finding is particularly important, as AOPP plays a role in stimulating inflammatory cytokine synthesis and release, a process that contributes to chronic inflammation in DM [48].

8-Hydroxydeoxyguanosine (8-OHdG) is a modified nucleoside formed when ROS oxidize guanine, one of the four nucleobases in DNA. This oxidative modification results in a structurally altered guanine derivative that is prone to cause mutations and genomic instability. Higher plasma flavonoid levels have been reported to correlate with lower 8-OHdG concentrations [49]. The LSEE treatments decreased 8-OHdG, and the mechanism may be the significant content of flavonoids.

Nitric oxide (NO), a highly reactive and unstable radical, functions as an essential signaling molecule for various physiological processes at low concentrations. Hyperglycemia in DM rapidly elevates iNOS gene expression, leading to excessive NO production. This overproduction, through its interaction with superoxide anions (O2-), facilitates the formation of peroxynitrite (ONOO-), a highly damaging RNS, significantly contributing to nitrosative stress and the progression of oxidative damage. Reactive peroxynitrite further leads to protein tyrosine nitration, causing structural alterations and cellular disruption. 3-nitrotyrosine (3NT), a stable nitrated protein, is, therefore, utilized as a biomarker for quantifying oxidative and nitrosative stress alongside other indicators [50,51]. LSEE treatment significantly reduced 3NT levels, demonstrating superior effectiveness compared to metformin and TX administration.

Advanced glycation end products (AGEs) are key contributors to the pathogenesis of DM and its complications, forming through the non-enzymatic glycation of proteins, lipids, and nucleic acids. Their accumulation is accelerated in hyperglycemia, leading to oxidative stress, chronic inflammation, and vascular dysfunction. Additionally, they impair pancreatic β-cell function, exacerbating hyperglycemia and perpetuating metabolic dysfunction. AGEs exert their effects primarily through interaction with the receptor for advanced glycation end products (RAGE), triggering ROS production, NF-κB activation, and pro-inflammatory cytokine release [52]. LSEE administration exhibited inhibitory activity on AGE formation, with effects comparable to those of metformin.

Antioxidants are essential for neutralizing ROS and maintaining redox homeostasis. Thiol (-SH) groups, particularly those found in cysteine residues and glutathione (GSH), are critical indicators of antioxidant capacity due to their ability to donate electrons and neutralize ROS. Plasma SHs are major circulating antioxidants, and their protection by natural antioxidants like flavonoids, ellagic acid, and quercetin has been previously documented [53]. In this study, plasma SH levels were significantly decreased in diabetic rats, indicating oxidative protein damage and depletion of antioxidant defenses. LSEE treatment at all tested concentrations significantly increased thiol levels, demonstrating comparable efficacy to TX and metformin.

Persistent oxidative stress activates nuclear factor kappa B (NF-κB) and other inflammatory pathways, leading to systemic inflammation, a key contributor to IR and metabolic dysfunction. Inflammation amplifies ROS production, and hyperglycemia concurrently drives chronic inflammation, establishing a self-sustaining cycle of inflammation, OS, and hyperglycemia. Consequently, interrupting this cycle is a primary therapeutic goal in diabetes management [54].

The nuclear factor-kappa B (NF-κB) family of transcription factors plays a key role in regulating inflammation and immune responses by controlling gene expression. The p65:p50 heterodimer is the most common form of NF-κB activated via the canonical pathway. When exposed to pro-inflammatory signals, NF-κB dimers translocate to the nucleus, promoting the transcription of inflammation-related genes. Antioxidants normally regulate ROS-induced NF-κB activation [55]. In DM, excess ROS causes sustained NF-κB activation and inflammation. ROS-induced DNA damage also contributes to this pathological NF-κB activation, intensifying inflammatory responses. Plant polyphenols act as both antioxidants and anti-inflammatory agents by inhibiting NF-κB activation, thereby helping to counteract oxidative stress and inflammation in DM [56]. In STZ-induced T1D rats LSEE managed to reduce NF-kB p65 only in 100% concentration, metformin and TX administration having a superior effect.

The NLRP3 inflammasome plays a critical role in chronic inflammation and metabolic dysfunction in DM, contributing to IR, β-cell dysfunction, and diabetic complications. Its activation occurs in two stages: priming and activation. The priming phase, triggered by hyperglycemia, oxidative stress, and lipotoxicity, activates NF-κB signaling, leading to increased NLRP3 expression and the production of pro-IL-1β and pro-IL-18. The activation phase is triggered by mitochondrial ROS production, endoplasmic reticulum (ER) stress, ATP release, potassium efflux, and AGEs-RAGE signaling, resulting in inflammasome assembly, caspase-1 activation, and the maturation of IL-1β and IL-18. A crucial downstream effector of inflammasome activation is Gasdermin D (GSDMD), which mediates pyroptosis, a form of inflammatory cell death. Upon caspase-1 cleavage, GSDMD forms membrane pores, allowing the release of IL-1β and IL-18, exacerbating inflammation and tissue damage [56]. Persistent NLRP3-GSDMD activation contributes to pancreatic β-cell apoptosis, IR in adipose and liver tissues, and vascular inflammation, promoting complications such as diabetic nephropathy, retinopathy, neuropathy, and atherosclerosis [57]. Given its pivotal role in diabetes-associated inflammation, the NLRP3 inflammasome and Gasdermin D have emerged as therapeutic targets. Antioxidants like quercetin, resveratrol, and ellagic acid help reduce mitochondrial ROS, preventing excessive NLRP3–Gasdermin D activation [58]. Our study demonstrated that LSEE effectively inhibited the NLRP3 inflammasome in diabetic rats, as evidenced by significant reductions in IL-1β, IL-18, GSDMD, and caspase-1 levels compared to untreated diabetic controls. While metformin and TX also reduced IL-1β, IL-18, and gasdermin D, they did not significantly affect caspase-1 activity, suggesting that LSEE exerted a more comprehensive inhibitory effect on the NLRP3 inflammasome pathway.

In DM, dyslipidemia stems from a complex interplay of metabolic disturbances, significantly increasing the risk of cardiovascular complications. A key factor is insulin deficiency or resistance, which impairs the activity of lipoprotein lipase. Furthermore, hyperglycemia stimulates the liver to overproduce very-low-density lipoprotein (VLDL), a primary contributor to elevated TG. IR also promotes increased free fatty acid flux from adipose tissue to the liver, further fueling VLDL synthesis. Additionally, high glucose levels can glycate lipoproteins, altering their structure and function, which may disrupt cholesterol metabolism and contribute to increased TC. The TyG index, calculated from fasting triglyceride and glucose levels, serves as a simple and cost-effective surrogate marker for IR. Elevated TyG index values correlate with increased risk of T2DM cardiovascular disease, and non-alcoholic fatty liver disease, making it a valuable tool for risk prediction and clinical assessment in DM [59,60]. Our study revealed significant lipid dysregulation, with elevated TG and TC levels, and an increased TyG index, indicative of IR. LSEE treatment effectively reduced both TG and TC in a dose-dependent manner, with the highest dose showing the greatest efficacy. Notably, LSEE also lowered the TyG index, with the 50% concentration being most effective. In contrast, metformin and TX did not significantly alter the TyG index.

Liver injury is a major complication of DM, driven by hyperglycemia and IR, which drive a cascade of detrimental effects. In general DM, this often presents as non-alcoholic fatty liver disease (NAFLD), defined by excessive hepatic fat accumulation, oxidative stress, and chronic inflammation mediated by cytokines and the NLRP3 inflammasome, potentially progressing to fibrosis and cirrhosis. The STZ-induced DM model in rodents effectively mimics these pathological changes by rapidly inducing hepatic steatosis due to insulin deficiency, increasing oxidative stress from STZ and hyperglycemia-induced ROS, and triggering inflammatory responses [61,62]. In the STZ-induced DM group, AST and ALT levels were elevated, while the AST/ALT ratio was decreased, indicating the presence of liver injury. LSEE administration exhibited a protective effect, as it significantly reduced AST and ALT levels while increasing the AST/ALT ratio. Notably, lower doses of LSEE demonstrated the most pronounced hepatoprotective effects.

PCOS and DM are closely linked through their pathophysiological mechanisms that include IR, androgen excess, chronic inflammation, and OS. In T2DM, PCOS is primarily driven by peripheral IR, compensatory hyperinsulinemia, and dyslipidemia, creating a metabolic–hormonal cycle that increases diabetes risk [63]. In patients with T1DM delayed puberty, earlier onset of menopause, and a higher prevalence of hirsutism and PCOS features have been observed. In T1DM, the main mechanism involves exogenous insulin administration which bypasses hepatic metabolism raising systemic insulin levels, which will further increase ovarian steroid synthesis. Furthermore, insulin acts as a co-gonadotropin and also suppresses SHBG hepatic synthesis leading to increased levels of free testosterone [64]. Currently, several short-term treatments are available for PCOS management, including symptomatic treatment, lifestyle modification, ovulation induction, anti-androgen therapy, and treatment of metabolic disorders. Etiological treatment is still lacking, and these therapies are often associated with significant side effects [65,66]. Adjunctive natural therapies targeting underlying pathophysiological mechanisms are gaining recognition in PCOS management. Polyphenolic compounds and flavonoids exert pleiotropic effects on hormonal dysregulation in PCOS, targeting key endocrine abnormalities that contribute to its complex pathophysiology [67]. These phytochemicals modulate steroidogenesis through the inhibition of enzymes such as 17β-hydroxysteroid dehydrogenase and 5α-reductase, thereby attenuating hyperandrogenism. Furthermore, they interact with estrogen receptors, exhibiting selective estrogen receptor modulator (SERM) activity or modulating aromatase expression, influencing the estrogen/androgen balance and potentially restoring cyclical ovarian function. By enhancing insulin sensitivity via activation of insulin signaling pathways, they indirectly regulate androgen biosynthesis, which is often stimulated by hyperinsulinemia in PCOS. Moreover, evidence suggests that these compounds may modulate LH secretion and increase SHBG production, further contributing to the restoration of hormonal homeostasis [68,69,70].

OS plays a key role in PCOS pathogenesis, contributing to IR, hyperandrogenism, chronic inflammation, and ovarian dysfunction. Increased ROS production impairs insulin signaling, leading to hyperinsulinemia, which stimulates androgen overproduction, disrupting follicular development and ovulation [70,71]. In this study, PCOS in rats was linked to OS, as evidenced by elevated oxidative biomarkers (TOS, OSI, MDA, AOPP, 8-OHdG, 3NT and AGEs) and reduced antioxidant markers (TAC and SH). LSEE treatment exhibited antioxidant activity by significantly lowering TOS, OSI, MDA, AOPP, and 8-OHdG, while notably increasing TAC levels. Similarly, LSEE demonstrated antioxidant effects comparable to metformin, though its efficacy was lower than that of TX, highlighting its moderate but beneficial antioxidant potential.

OS-induced lipid peroxidation and mitochondrial dysfunction contribute to chronic low-grade inflammation by activating the NF-κB pathway, leading to increased production of pro-inflammatory cytokines (TNF-α, IL-6, IL-1β). Additionally, OS stimulates the NLRP3 inflammasome, further amplifying inflammatory responses. This inflammatory state promotes IR, which in turn drives androgen overproduction, follicular dysfunction, and anovulation. OS also induces damage to oocytes and granulosa cells, impairing fertility and endometrial receptivity. Furthermore, it disrupts adipose tissue homeostasis by increasing pro-inflammatory adipokines (resistin, leptin) while reducing adiponectin, thereby exacerbating metabolic disturbances in PCOS [72,73,74].

The findings of this study demonstrated that LET-induced PCOS was associated with elevated pro-inflammatory cytokine levels, confirming the activation of the NLRP3 inflammasome. LSEE administration significantly reduced NF-κB and NLRP3 activation, leading to a subsequent decrease in pro-inflammatory cytokines IL-1β and IL-18, as well as a no significant activity on the anti-inflammatory cytokine IL-10, indicating its regulatory effect on inflammation in PCOS. Additionally, the present study demonstrated that LSEE therapy effectively improved hormonal and metabolic imbalances, reduced oxidative stress and inflammation, and alleviated ultrasonographic and histomorphological abnormalities in ovarian and uterine tissues, highlighting its potential therapeutic benefits in PCOS management.

In our study, PCOS rats developed hormonal imbalances, including elevated gonadotropins, reduced estrogen levels, and increased testosterone levels. Ultrasound examination further revealed polycystic ovarian morphology, supporting the induction of PCOS. Additionally, metabolic disturbances were observed, with elevated insulin and glucose levels, along with an increased HOMA-IR index, indicating the presence of IR. LSEE treatment alleviated hormonal imbalances by reducing LH and FSH levels, increasing estrogen, and lowering testosterone levels, indicating its regulatory effect on the hypothalamic-pituitary-ovarian (HPO) axis. Additionally, LSEE treatment improved insulin sensitivity by reducing insulin levels and the HOMA-IR index, though its effect was modest compared to metformin. However, LSEE demonstrated superior efficacy in correcting hormonal disturbances when compared to metformin, suggesting a potential advantage in addressing endocrine dysfunction in PCOS. In PCOS rats significant ovarian histological changes were described, including cyst formation, atretic follicles, and a diminished corpus luteum, indicative of disrupted follicular development and anovulation. Treatment with LSEE and metformin effectively ameliorated these pathological alterations, reducing cyst formation, decreasing atretic follicles, and promoting corpus luteum formation, suggesting a potential restoration of normal follicular dynamics and ovulatory function.

This study presents several limitations, including a limited number of animals, a short intervention period, and reliance on a single animal model, which may affect the generalizability of the results to human contexts. Additionally, we did not conduct a separate toxicity assessment of the LSEE, which we will test include our future studies. Furthermore, employing only one extraction technique might have impacted the yield and variety of bioactive constituents in the plant extract. Another limitation of this study is that the phytochemical characterization of the LSEE was based on general spectrophotometric assays, and results were expressed as equivalents of standard compounds (e.g., gallic acid, ellagic, and luteolin equivalents). Future studies should incorporate advanced chromatographic techniques (e.g., LC-MS) for more detailed profiling and quantification of specific compounds within the extract. As a result, the effects observed in this study may not entirely reflect the full therapeutic potential of the *L. salicaria* L. species.

## 5. Conclusions

The findings of this study demonstrate that LSEE exhibits potential therapeutic efficacy in mitigating pathophysiological parameters associated with both DM and PCOS. LSEE demonstrated significant, dose-dependent antioxidant and anti-inflammatory effects in STZ-induced DM, with a clear positive correlation between dosage and efficacy. Additionally, LSEE effectively mitigated metabolic and hepatic dysfunction in STZ-induced T1D rats. In a separate investigation, utilizing the LET-induced PCOS rat model, LSEE exhibited therapeutic potential by modulating hormonal and metabolic parameters, and by reducing OS and inflammation, likely attributed to its polyphenolic constituents. While these findings suggest LSEE’s therapeutic utility, the potential for paradoxical pro-oxidant effects at elevated antioxidant concentrations necessitates careful dose optimization.

## Figures and Tables

**Table 1:** The content of phenolic compounds in LSEE by HPLC (μg/mL).

Peak No.	R_t_ (min)	UV λ_max_ (nm)	[M+H]^+^ (*m/z*)	Phenolic Compound	Subclass	LSEE
1	3.19	270	139	2-Hydroxybenzoic acid	Hydroxybenzoic acid	253.16 ± 9.32
2	9.03	260, 360	935	Castalagin	Ellagitannin	301.23 ± 15.26
3	10.56	260, 360	935	Vescalagin	Ellagitannin	537.53 ± 30.62
4	11.99	277	485	Digalloyl-glucoside	Gallotanin	231.69 ± 5.78
5	13.54	260	465, 303	Ellagic acid-glucoside	Hydroxybenzoic acid	288.59 ± 6.44
6	14.65	350, 260	449, 287	Luteolin-glucoside	Flavone	208.94 ± 5.03
7	15.03	350, 260	449, 287	Luteolin-glucoside isomer	Flavone	133.21 ± 2.38
8	15.93	340, 270	433, 271	Apigenin-glucoside	Flavone	140.85 ± 4.55
9	16.37	260	303	Ellagic acid	Hydroxybenzoic acid	326.17 ± 11.92
10	18.38	260	792, 303	Ellagic acid derivative	Hydroxybenzoic acid	249.406 ± 12.51
11	23.37	350, 260	287	Luteolin	Flavone	58.39 ± 1.22
				Total phenolics		2729.168 ± 139.48

Quantity was expressed as μg gallic acid equivalent/mL for hydroxybenzoic acids; μg ellagic acid equivalent/mL for ellagitannins; μg luteolin equivalent/mL for flavones.

**Table 2:** The in vitro antioxidant test results in *L. salicaria* L. ethanolic extract.

Sample	DPPH (μgTE/mL)	H_2_O_2_ Scavenging Activity (μgTE/mL)	NO Scavenging Activity (μgQE/mL)	FRAP (mgTE/mL)
***L. salicaria* IC50**	79.84 ± 8.22	107.05 ± 12.03	27.51 ± 2.56	313.82 ± 39.82
**Trolox IC50**	11.2 ± 1.7	24.23 ± 3.12	-	12.07 ± 2.04
**Quercetin IC50**	-	-	20.58 ± 3.67	-
***p*-value**	<0.001	<0.001	<0.01	<0.001

DPPH—α, α-diphenyl-β-picrylhydrazyl; NO—nitric oxide; H_2_O_2_—hydrogen peroxide; FRAP—Ferric reducing antioxidant power; QE—quercetin equivalent; TE—Trolox equivalent.

**Table 3:** Effects of *L. salicaria* ethanol extract on inflammatory markers in rat STZ-induced DM.

Parameters	Control	STZ	STZ + LSEE 100%	STZ + LSEE 50%	STZ + LSEE 25%	STZ + M	STZ + TX
**Gasdermin (ng/mL)**	5.87 ± 1.05	9.50 ± 1.65 ^aa^	0.28 ± 0.05 ^bbb,ccc,ddd^	0.93 ± 0.05 ^bbb,ccc,ddd^	0.34 ± 0.05 ^bbb,ccc,ddd^	9.38 ± 1.11	6.52 ± 1.35 ^b^
**IL-18 (pg/mL)**	16.30 ± 9.86	59.45 ± 8.49 ^aa^	16.19 ± 3.70 ^bbb,ccc^	12.69 ± 5.18 ^bbb,ccc,^	14.30 ± 2.33 ^bbb,ccc^	37.29 ± 6.97 ^bb^	11.74 ± 4.59 ^bbb^
**Caspase (pg/mL)**	37.03 ± 7.11	39.49 ± 2.14	35.18 ± 5.42	30.53 ± 5.39 ^b,c^	20.83 ± 7.13 ^bbb,cc^	51.63 ± 21.77	42.19 ± 8.81
**NFkB-p65 (pg/mL)**	165.11 ± 16.21	306.33 ± 28.07 ^aaa^	255.24 ± 83.59	487.87 ± 69.34 ^bbb,ccc,ddd^	509.93 ± 98.29 ^bb,ccc,ddd^	230.55 ± 18.46 ^bb^	222.58 ± 21.94 ^bb^
**IL-1β (pg/mL)**	23.75 ± 3.15	132.50 ± 21.48 ^aaa^	33.71 ± 5.99 ^bbb,cc^	23.88 ± 2.46 ^bbb,c^	32.42 ± 5.63 ^bbb,cc^	19.58 ± 3.99 ^bbb^	27.92 ± 3.15 ^bbb^

Values are expressed as mean ± SD (standard deviation). ^a^ vs. CONTROL: ^aa^ *p* < 0.01, ^aaa^ *p* < 0.001; ^b^ vs. STZ: ^b^ *p* < 0.05, ^bb^ *p* < 0.01, ^bbb^ *p* < 0.001; ^c^ vs. STZ + M: ^c^ *p* < 0.05, ^cc^ *p* < 0.01, ^ccc^ *p* < 0.001; ^d^ vs. STZ + TX: ^ddd^ *p* < 0.001; STZ—STZ; STZ + TX—STZ + Trolox; STZ + M—STZ + Metformin; LSEE—*L. salicaria* L. ethanol extract; NFkB-p65—Nuclear Factor Kappa B-p65 subunit; IL-1β—Interleukin-1 Beta; IL-18—Interleukin-18.

**Table 4:** Effects of *L. salicaria* L. ethanol extract on inflammatory markers in rat Letrozole-induced PCOS.

Parameters	Control	LET	LET + LSEE100%	LET + M	LET + TX
**NFkB-p65 (pg/mL)**	258.95 ± 48.74	521.69 ± 75.02 ^aa^	341.65 ± 58.01 ^bb,cc^	541.67 ± 94.76	269.08 ± 90.45 ^b^
**IL-1β (pg/mL)**	24.75 ± 1.73	44.82 ± 11.83 ^aa^	18.68 ± 2.63 ^bb^	21.92 ± 3.21 ^bb^	26.45 ± 1.30 ^bb^
**IL-18 (pg/mL)**	19.50 ± 1.80	34.16 ± 7.30 ^aa^	24.16 ± 8.95	15.39 ± 2.22 ^bbb^	21.35 ± 6.50 ^b^
**IL-10 (ng/mL)**	15.27 ± 2.15	29.48 ± 5.45 ^aaa^	23.35 ± 2.45 ^b,cc^	14.90 ± 1.73 ^bbb^	22.67 ± 1.74 ^b^

Values are expressed as mean ± SD (standard deviation). ^a^ vs. CONTROL: ^aa^ *p* < 0.01, ^aaa^ *p* < 0.001; ^b^ vs. STZ: ^b^ *p* < 0.05, ^bb^ *p* < 0.01, ^bbb^ *p* < 0.001; ^c^ vs. LET + M: ^cc^ *p* < 0.01; LET—Letrozole; LET + TX—Letrozole + Trolox; LET + M—Letrozole + Metformin; LSEE—*L. salicaria* L. ethanol extract; NFkB-p65—Nuclear Factor Kappa B-p65 subunit; IL-1β—Interleukin-1 Beta; IL-18—Interleukin-18.

**Table 5:** Effects of *L. salicaria* L. on hormone profiles in rat Letrozole-induced PCOS.

Parameters	Control	LET	LET + LSEE 100%	LET + M	LET + TX
**Insulin (pg/mL)**	30.55 ± 5.70	98.72 ± 11.32 ^aaa^	88.91 ± 9.87 ^ccc,ddd^	25.91 ± 4.80 ^bb^	27.90 ± 8.5 ^bb^
**HOMA-IR**	6.64 ± 0.53	31.93 ± 4.50	20.78 ± 1.98 ^bb,ccc.ddd^	5.08 ± 0.56 ^bbb^	7.16 ± 0.74 ^bbb^
**GLU (mg/dL)**	90.43 ± 15.56	131.52 ± 14.09	95.25 ± 11.35 ^bb^	80.4 ± 11.31 ^bb^	111.73 ± 35.21
**FSH (pg/mL)**	39.80 ± 5.74	99.96 ± 31.89 ^aa^	51.08 ± 3.05 ^b,ccc,ddd^	99.39 ± 3.61	101.15 ± 34.59
**LH (pg/mL)**	9.81 ± 4.37	25.08 ± 8.22 ^aa^	17.51 ± 2.91 ^bbb^	20.62 ± 4.04 ^bbb^	13.11 ± 2.95 ^bbb^
**Estrogen (pg/mL)**	596.40 ± 66.99	329.78 ± 55.13 ^aaa^	629.57 ± 25.87 ^bbb,cc^	774.88 ± 55.77 ^bbb^	622.02 ± 166.26 ^bbb^
**Testosterone (ng/mL)**	0.94 ± 0.28	1.60 ± 0.86 ^a^	0.82 ± 0.07 ^b,dd^	1.51 ± 0.99	2.36 ± 0.81

Values are expressed as mean ± SD (standard deviation). ^a^ vs. CONTROL: ^a^ *p* < 0.05, ^aa^ *p* < 0.01, ^aaa^ *p* < 0.001; ^b^ vs. LET: ^b^ *p* < 0.05, ^bb^ *p* < 0.01, ^bbb^ *p* < 0.001; ^c^ vs. LET + M: ^cc^ *p* < 0.01, ^ccc^ *p* < 0.001; ^d^ vs. LET + TX: ^dd^ *p* < 0.01, ^ddd^ *p* < 0.001; LET—Letrozole; LSEE—*L. salicaria* L. ethanol extract; TX—Trolox; M—Metformin.

## Data Availability

Data are available only for reviewers until the first author defends her Ph.D. thesis.

## References

[1] Piwowarski J.P., Granica S., Kiss A.K. (2015). *Lythrum salicaria* L.-Underestimated medicinal plant from European traditional medicine. A review. J. Ethnopharmacol..

[2] Mocan A., Vlase L., Vodnar D.C., Bischin C., Hanganu D., Gheldiu A.M., Oprean R., Silaghi-Dumitrescu R., Crișan G. (2014). Polyphenolic content, antioxidant and antimicrobial activities of Lycium barbarum L. and Lycium chinense Mill. leaves. Molecules.

[3] Savran A., Zengin G., Aktumsek A., Mocan A., Glamoćlija J., Ćirić A., Soković M. (2016). Phenolic compounds and biological effects of edible Rumex scutatus and Pseudosempervivum sempervivum: Potential sources of natural agents with health benefits. Food Funct..

[4] Babotă M., Mocan A., Vlase L., Crișan O., Ielciu I., Gheldiu A.M., Vodnar D.C., Crișan G., Păltinean R. (2018). Phytochemical Analysis, Antioxidant and Antimicrobial Activities of *Helichrysum arenarium* (L.) Moench. and *Antennaria dioica* (L.) Gaertn. Flowers. Molecules.

[5] Erel O. (2005). A new automated colorimetric method for measuring total oxidant status. Clin. Biochem..

[6] Erel O. (2004). A novel automated method to measure total antioxidant response against potent free radical reactions. Clin. Biochem..

[7] Patel A., Patel A., Patel A., Patel N.M. (2010). Determination of polyphenols and free radical scavenging activity of *Tephrosia purpurea* linn leaves (Leguminosae). Pharmacognosy Res..

[8] Al-Amiery A.A., Al-Majedy Y.K., Kadhum A.A., Mohamad A.B. (2015). Hydrogen Peroxide Scavenging Activity of Novel Coumarins Synthesized Using Different Approaches. PLoS ONE.

[9] Ghasemi A., Jeddi S. (2023). Streptozotocin as a tool for induction of rat models of diabetes: A practical guide. Excli J..

[10] But A.E., Pop R.M., Binsfeld G.F., Ranga F., Orăsan M.S., Cecan A.D., Morar I.I., Chera E.I., Bonci T.I., Usatiuc L.O. (2024). The Phytochemical Composition and Antioxidant Activity of Matricaria recutita Blossoms and Zingiber officinale Rhizome Ethanol Extracts. Nutrients.

[11] Calco G.N., Proskocil B.J., Jacoby D.B., Fryer A.D., Nie Z. (2021). Metformin prevents airway hyperreactivity in rats with dietary obesity. Am. J. Physiol. Lung Cell Mol. Physiol..

[12] Ragy M.M., Abdel-Hamid H.A., Toni N.D.M. (2019). Pathophysiological changes in experimental polycystic ovary syndrome in female albino rats: Using either hemin or L-arginine. J. Cell Physiol..

[13] Harma M., Harma M., Erel O. (2003). Increased oxidative stress in patients with hydatidiform mole. Swiss Med. Wkly..

[14] Essel L.B., Obiri D.D., Osafo N., Antwi A.O., Duduyemi B.M. (2017). The Ethanolic Stem-Bark Extract of Antrocaryon micraster Inhibits Carrageenan-Induced Pleurisy and Pedal Oedema in Murine Models of Inflammation. Int. Sch. Res. Notices.

[15] Miranda K.M., Espey M.G., Wink D.A. (2001). A rapid, simple spectrophotometric method for simultaneous detection of nitrate and nitrite. Nitric Oxide.

[16] Tsikas D. (2017). Assessment of lipid peroxidation by measuring malondialdehyde (MDA) and relatives in biological samples: Analytical and biological challenges. Anal. Biochem..

[17] Witko-Sarsat V., Friedlander M., Capeillère-Blandin C., Nguyen-Khoa T., Nguyen A.T., Zingraff J., Jungers P., Descamps-Latscha B. (1996). Advanced oxidation protein products as a novel marker of oxidative stress in uremia. Kidney Int..

[18] Laragione T., Gianazza E., Tonelli R., Bigini P., Mennini T., Casoni F., Massignan T., Bonetto V., Ghezzi P. (2006). Regulation of redox-sensitive exofacial protein thiols in CHO cells. Biol. Chem..

[19] Nabipoorashrafi S.A., Seyedi S.A., Rabizadeh S., Ebrahimi M., Ranjbar S.A., Reyhan S.K., Meysamie A., Nakhjavani M., Esteghamati A. (2022). The accuracy of triglyceride-glucose (TyG) index for the screening of metabolic syndrome in adults: A systematic review and meta-analysis. Nutr. Metab. Cardiovasc. Dis..

[20] Pop R.M., Vassilopoulou E., Jianu M.E., Roșian Ș.H., Taulescu M., Negru M., Bercian C., Boarescu P.M., Bocsan I.C., Feketea G. (2024). Nigella sativa oil attenuates inflammation and oxidative stress in experimental myocardial infarction. BMC Complement. Med. Ther..

[21] Sumitra M., Manikandan P., Rao K.V., Nayeem M., Manohar B.M., Puvanakrishnan R. (2004). Cardiorespiratory effects of diazepam-ketamine, xylazine-ketamine and thiopentone anesthesia in male Wistar rats—A comparative analysis. Life Sci..

[22] Manayi A., Khanavi M., Saeidnia S., Azizi E., Mahmoodpour M.R., Vafi F., Malmir M., Siavashi F., Hadjiakhoondi A. (2013). Biological activity and microscopic characterization of *Lythrum salicaria* L. Daru.

[23] Lamela M., Cadavid I., Calleja J.M. (1986). Effects of Lythrum salicaria extracts on hyperglycemic rats and mice. J. Ethnopharmacol..

[24] Bencsik T., Horváth G., Papp N. (2011). Variability of total flavonoid, polyphenol and tannin contents in some Lythrum salicaria populations. Nat. Prod. Commun..

[25] Granica S., Vahjen W., Zentek J., Melzig M.F., Pawłowska K.A., Piwowarski J.P. (2020). Lythrum salicaria Ellagitannins Stimulate IPEC-J2 Cells Monolayer Formation and Inhibit Enteropathogenic *Escherichia coli* Growth and Adhesion. J. Nat. Prod..

[26] Guo S., Ren X., He K., Chen X., Zhang S., Roller M., Zheng B., Zheng Q., Ho C.T., Bai N. (2020). The anti-diabetic effect of eight Lagerstroemia speciosa leaf extracts based on the contents of ellagitannins and ellagic acid derivatives. Food Funct..

[27] Tang B., Chen G.X., Liang M.Y., Yao J.P., Wu Z.K. (2015). Ellagic acid prevents monocrotaline-induced pulmonary artery hypertension via inhibiting NLRP3 inflammasome activation in rats. Int. J. Cardiol..

[28] Shen S.C., Chang W.C. (2013). Hypotriglyceridemic and hypoglycemic effects of vescalagin from Pink wax apple [*Syzygium samarangense* (Blume) Merrill and Perry cv. Pink] in high-fructose diet-induced diabetic rats. Food Chem..

[29] Polce S.A., Burke C., França L.M., Kramer B., de Andrade Paes A.M., Carrillo-Sepulveda M.A. (2018). Ellagic Acid Alleviates Hepatic Oxidative Stress and Insulin Resistance in Diabetic Female Rats. Nutrients.

[30] Ghadimi M., Foroughi F., Hashemipour S., Nooshabadi M.R., Ahmadi M.H., Yari M.G., Kavianpour M., Haghighian H.K. (2021). Decreased insulin resistance in diabetic patients by influencing Sirtuin1 and Fetuin-A following supplementation with ellagic acid: A randomized controlled trial. Diabetol. Metab. Syndr..

[31] Kilic I., Yeşiloğlu Y., Bayrak Y. (2014). Spectroscopic studies on the antioxidant activity of ellagic acid. Spectrochim. Acta A Mol. Biomol. Spectrosc..

[32] Mehrzadi S., Mehrabani M., Malayeri A.R., Bakhshayesh M., Kalantari H., Goudarzi M. (2019). Ellagic acid as a potential antioxidant, alleviates methotrexate-induced hepatotoxicity in male rats. Acta Chir. Belg..

[33] Gil T.Y., Hong C.H., An H.J. (2021). Anti-Inflammatory Effects of Ellagic Acid on Keratinocytes via MAPK and STAT Pathways. Int. J. Mol. Sci..

[34] Bains M., Kaur J., Akhtar A., Kuhad A., Sah S.P. (2022). Anti-inflammatory effects of ellagic acid and vanillic acid against quinolinic acid-induced rat model of Huntington’s disease by targeting IKK-NF-κB pathway. Eur. J. Pharmacol..

[35] Kahksha, Alam O., Al-Keridis L.A., Khan J., Naaz S., Alam A., Ashraf S.A., Alshammari N., Adnan M., Beg M.A. (2023). Evaluation of Antidiabetic Effect of Luteolin in STZ Induced Diabetic Rats: Molecular Docking, Molecular Dynamics, In Vitro and In Vivo Studies. J. Funct. Biomater..

[36] Bai L., Li X., He L., Zheng Y., Lu H., Li J., Zhong L., Tong R., Jiang Z., Shi J. (2019). Antidiabetic Potential of Flavonoids from Traditional Chinese Medicine: A Review. Am. J. Chin. Med..

[37] Liu H., Huang P., Wang X., Ma Y., Tong J., Li J., Ding H. (2024). Apigenin analogs as α-glucosidase inhibitors with antidiabetic activity. Bioorg Chem..

[38] Chagas M., Behrens M.D., Moragas-Tellis C.J., Penedo G.X.M., Silva A.R., Gonçalves-de-Albuquerque C.F. (2022). Flavonols and Flavones as Potential anti-Inflammatory, Antioxidant, and Antibacterial Compounds. Oxid. Med. Cell Longev..

[39] Wu Q., Li W., Zhao J., Sun W., Yang Q., Chen C., Xia P., Zhu J., Zhou Y., Huang G. (2021). Apigenin ameliorates doxorubicin-induced renal injury via inhibition of oxidative stress and inflammation. Biomed. Pharmacother..

[40] Yang Y., Liu M., Liu C., Tang S., Gu D., Tian J., Huang D., He F. (2023). Ellagic acid from pomegranate peel: Consecutive countercurrent chromatographic separation and antioxidant effect. Biomed. Chromatogr..

[41] Tunalier Z., Koşar M., Küpeli E., Çaliş İ., Başer K.H.C. (2007). Antioxidant, anti-inflammatory, anti-nociceptive activities and composition of *Lythrum salicaria* L. extracts. J. Ethnopharmacol..

[42] Eleazu C.O., Eleazu K.C., Chukwuma S., Essien U.N. (2013). Review of the mechanism of cell death resulting from streptozotocin challenge in experimental animals, its practical use and potential risk to humans. J. Diabetes Metab. Disord..

[43] Usatiuc L.O., Pârvu M., Pop R.M., Uifălean A., Vălean D., Szabo C.E., Țicolea M., Cătoi F.A., Ranga F., Pârvu A.E. (2024). Phytochemical Profile and Antidiabetic, Antioxidant, and Anti-Inflammatory Activities of *Gypsophila paniculata* Ethanol Extract in Rat Streptozotocin-Induced Diabetes Mellitus. Antioxidants.

[44] Darenskaya M.A., Kolesnikova L.I., Kolesnikov S.I. (2021). Oxidative Stress: Pathogenetic Role in Diabetes Mellitus and Its Complications and Therapeutic Approaches to Correction. Bull. Exp. Biol. Med..

[45] Dos Santos J.M., Zhong Q., Benite-Ribeiro S.A., Heck T.G. (2023). New Insights into the Role of Oxidative Stress in the Development of Diabetes Mellitus and Its Complications. J. Diabetes Res..

[46] Dong H., Sun Y., Nie L., Cui A., Zhao P., Leung W.K., Wang Q. (2024). Metabolic memory: Mechanisms and diseases. Signal Transduct. Target. Ther..

[47] Brunelli E., La Russa D., Pellegrino D. (2017). Impaired Oxidative Status Is Strongly Associated with Cardiovascular Risk Factors. Oxid. Med. Cell Longev..

[48] Kaczmarczyk-Sedlak I., Folwarczna J., Sedlak L., Zych M., Wojnar W., Szumińska I., Wyględowska-Promieńska D., Mrukwa-Kominek E. (2019). Effect of caffeine on biomarkers of oxidative stress in lenses of rats with streptozotocin-induced diabetes. Arch. Med. Sci..

[49] Urbaniak S.K., Boguszewska K., Szewczuk M., Kaźmierczak-Barańska J., Karwowski B.T. (2020). 8-Oxo-7,8-Dihydro-2′-Deoxyguanosine (8-oxodG) and 8-Hydroxy-2′-Deoxyguanosine (8-OHdG) as a Potential Biomarker for Gestational Diabetes Mellitus (GDM) Development. Molecules.

[50] Tessari P., Cecchet D., Cosma A., Vettore M., Coracina A., Millioni R., Iori E., Puricelli L., Avogaro A., Vedovato M. (2010). Nitric oxide synthesis is reduced in subjects with type 2 diabetes and nephropathy. Diabetes.

[51] Gutiérrez-Camacho L.R., Kormanovski A., Del Carmen Castillo-Hernández M., Guevara-Balcázar G., Lara-Padilla E. (2020). Alterations in glutathione, nitric oxide and 3-nitrotyrosine levels following exercise and/or hyperbaric oxygen treatment in mice with diet-induced diabetes. Biomed. Rep..

[52] Khalid M., Petroianu G., Adem A. (2022). Advanced Glycation End Products and Diabetes Mellitus: Mechanisms and Perspectives. Biomolecules.

[53] Balcerczyk A., Grzelak A., Janaszewska A., Jakubowski W., Koziol S., Marszalek M., Rychlik B., Soszynski M., Bilinski T., Bartosz G. (2003). Thiols as major determinants of the total antioxidant capacity. Biofactors.

[54] Baker R.G., Hayden M.S., Ghosh S. (2011). NF-κB, inflammation, and metabolic disease. Cell Metab..

[55] Liu T., Zhang L., Joo D., Sun S.-C. (2017). NF-κB signaling in inflammation. Signal Transduct. Target. Ther..

[56] Zhu L., Han J., Yuan R., Xue L., Pang W. (2018). Berberine ameliorates diabetic nephropathy by inhibiting TLR4/NF-κB pathway. Biol. Res..

[57] Ding S., Xu S., Ma Y., Liu G., Jang H., Fang J. (2019). Modulatory Mechanisms of the NLRP3 Inflammasomes in Diabetes. Biomolecules.

[58] Jin Q., Liu T., Qiao Y., Liu D., Yang L., Mao H., Ma F., Wang Y., Peng L., Zhan Y. (2023). Oxidative stress and inflammation in diabetic nephropathy: Role of polyphenols. Front. Immunol..

[59] Chillarón J.J., Sales M.P., Flores Le-Roux J.A., Castells I., Benaiges D., Sagarra E., Pedro-Botet J. (2013). Atherogenic dyslipidemia in patients with type 1 diabetes mellitus. Med. Clin..

[60] Zhang L., Zeng L. (2023). Non-linear association of triglyceride-glucose index with prevalence of prediabetes and diabetes: A cross-sectional study. Front. Endocrinol..

[61] Targher G., Corey K.E., Byrne C.D., Roden M. (2021). The complex link between NAFLD and type 2 diabetes mellitus—Mechanisms and treatments. Nat. Rev. Gastroenterol. Hepatol..

[62] Stefan N., Cusi K. (2022). A global view of the interplay between non-alcoholic fatty liver disease and diabetes. Lancet Diabetes Endocrinol..

[63] Teck J. (2022). Diabetes-Associated Comorbidities. Prim. Care.

[64] Escobar-Morreale H.F., Roldán-Martín M.B. (2016). Type 1 Diabetes and Polycystic Ovary Syndrome: Systematic Review and Meta-analysis. Diabetes Care.

[65] Di Lorenzo M., Cacciapuoti N., Lonardo M.S., Nasti G., Gautiero C., Belfiore A., Guida B., Chiurazzi M. (2023). Pathophysiology and Nutritional Approaches in Polycystic Ovary Syndrome (PCOS): A Comprehensive Review. Curr. Nutr. Rep..

[66] Liao B., Qiao J., Pang Y. (2021). Central Regulation of PCOS: Abnormal Neuronal-Reproductive-Metabolic Circuits in PCOS Pathophysiology. Front. Endocrinol..

[67] Malik S., Saeed S., Saleem A., Khan M.I., Khan A., Akhtar M.F. (2023). Alternative treatment of polycystic ovary syndrome: Pre-clinical and clinical basis for using plant-based drugs. Front. Endocrinol..

[68] Alesi S., Ee C., Moran L.J., Rao V., Mousa A. (2022). Nutritional Supplements and Complementary Therapies in Polycystic Ovary Syndrome. Adv. Nutr..

[69] Manouchehri A., Abbaszadeh S., Ahmadi M., Nejad F.K., Bahmani M., Dastyar N. (2023). Polycystic ovaries and herbal remedies: A systematic review. JBRA Assist. Reprod..

[70] Cappelli V., Musacchio M.C., Bulfoni A., Morgante G., De Leo V. (2017). Natural molecules for the therapy of hyperandrogenism and metabolic disorders in PCOS. Eur. Rev. Med. Pharmacol. Sci..

[71] Macut D., Bjekić-Macut J., Savić-Radojević A. (2013). Dyslipidemia and oxidative stress in PCOS. Front. Horm. Res..

[72] Armanini D., Boscaro M., Bordin L., Sabbadin C. (2022). Controversies in the Pathogenesis, Diagnosis and Treatment of PCOS: Focus on Insulin Resistance, Inflammation, and Hyperandrogenism. Int. J. Mol. Sci..

[73] Orisaka M., Mizutani T., Miyazaki Y., Shirafuji A., Tamamura C., Fujita M., Tsuyoshi H., Yoshida Y. (2023). Chronic low-grade inflammation and ovarian dysfunction in women with polycystic ovarian syndrome, endometriosis, and aging. Front. Endocrinol..

[74] Zhai Y., Pang Y. (2022). Systemic and ovarian inflammation in women with polycystic ovary syndrome. J. Reprod. Immunol..

